# Oleic acid from cancer-associated fibroblast promotes cancer cell stemness by stearoyl-CoA desaturase under glucose-deficient condition

**DOI:** 10.1186/s12935-022-02824-3

**Published:** 2022-12-13

**Authors:** Sung-Hyun Hwang, Yeseul Yang, Jae-Ha Jung, Yongbaek Kim

**Affiliations:** 1 Laboratory of Clinical Pathology, College of Veterinary Medicine, Seoul National University, 1 Gwanak-Ro, Gwanak-Gu, Seoul, 08826 Republic of Korea; 2 BK21 Future Veterinary Medicine Leading Education and Research Center, College of Veterinary Medicine, Seoul National University, 1 Gwanak-Ro, Gwanak-Gu, Seoul, 08826 Republic of Korea; 3 Laboratory of Clinical Pathology, Research Institute for Veterinary Science, College of Veterinary Medicine, Seoul National University, 1 Gwanak-Ro, Gwanak-Gu, Seoul, 08826 Republic of Korea; 4 Biomedical Research Institute, Seoul National University Bundang Hospital, Seongnam, 13620 Republic of Korea

**Keywords:** Cancer-associated fibroblasts, Oleic acid, Stearoyl-CoA desaturase, Glucose starvation, Lipid metabolism

## Abstract

**Background:**

Cancer-associated fibroblasts (CAFs) coordinate the malignancy of cancer cells via secretory materials. Reprogrammed lipid metabolism and signaling play critical roles in cancer biology. Oleic acid (OA) serves as a source of energy under glucose-deficient conditions, but its function in cancer progression remains unclear. The present study investigated that CAFs in xenografted tumors had higher amounts of fatty acids, particularly OA, compared to normal fibroblasts, and promoted the cancer cell stemness in lung adenocarcinoma cells under glucose-deficient condition.

**Methods:**

Xenografts were established in immunodeficient mice by injection of NCI-H460 (H460) cells. Lipids and fatty acids were evaluated using the BODIPY staining and fatty-acid methyl esters analysis. The expression levels of markers for lipid metabolism and cancer stemness were determined by western blot, flow cytometry, and real-time PCR. Cancer cell subclones against stearoyl-CoA desaturase (SCD) were produced by lentiviral vector and CRISPR/cas9 systems. The expression of SCD was examined immunochemically in human adenocarcinoma tissues, and its clinical relevance to survival rate in lung adenocarcinoma patients was assessed by Kaplan–Meier analysis.

**Results:**

Transferred CAF-derived OA through lipid transporter upregulated SCD in cancer cells under glucose-deficient conditions, resulting in enhanced lipid metabolism and autophagosome maturation. By OA treatment under glucose deficient condition, cancer cell stemness was significantly enhanced through sequential activation of SCD, F-actin polymerization and nuclear translocation of yes-associated protein. These findings were confirmed by experiments using chemical inhibitors, SCD-overexpressing cells and SCD-knockout (KO) cells. When xenografted, SCD-overexpressing cells produced larger tumors compared with parental cells, while SCD-KO cells generated much smaller tumors. Analysis of tumor tissue microarray from lung adenocarcinoma patients revealed that SCD expression was the marker for poor prognosis involving tumor grade, clinical stage and survival rate.

**Conclusion:**

Our data indicate that CAFs-derived OA activated lipid metabolism in lung adenocarcinoma cells under glucose-deficient conditions, subsequently enhancing stemness and progression toward malignancy.

**Supplementary Information:**

The online version contains supplementary material available at 10.1186/s12935-022-02824-3.

## Background

A hostile microenvironment by cancer cell is established by abnormal angiogenesis and affects the metabolic profile in the tumor tissue [1, 2]. Cancer cells consume high amounts of glucose and oxygen to produce ATP, resulting in glucose deficiency. Glucose starvation confers metabolic reprogramming in cancer cells via dysregulated AMPK expression [3]. For example, to survive under glucose-deficient condition, the cancer cells activate the lipid metabolism including FA desaturation and β-oxidation for the production of energy [4]. Alternatively, lipids-mediated autophagy provides an energy source during glucose starvation [5, 6]. Stearoyl-CoA desaturase (SCD) is essential for the earliest step of autophagosome formation that supports the undisturbed growth of cancer cells [7]. High expression of SCD is associated with poor prognosis and metastasis in patients with colorectal cancer [8].

Cancer-associated fibroblasts (CAFs) modulate the microenvironment around cancer cells. They produce extracellular matrix and interact with cancer cells via secretory materials [9]. In tumor masses, various molecules are biosynthesized in CAFs, affecting cancer cell phenotypes [10, 11]. Moreover, CAFs supplement the metabolic needs of cancer cells by providing essential nutrients and energy-rich metabolites [12]. Direct transfer of CAF-derived metabolites to cancer cells increases the mitochondrial activity via upregulation of SIRT1-dependent peroxisome proliferator-activated receptor gamma coactivator 1-α [13]. Exogeneous lipid is internalized through endocytosis, diffusion, and receptor on lipid raft of the plasma membrane. Uptake of long-chain fatty acid is dependent on CD36, a lipid transporter, but not endocytosis [14].

Oleic acid (OA), a monounsaturated fatty acid (MUFA), is essential to produce energy by activation of β-oxidation in cancer cells [15]. OA is important to maintain the cellular membrane. OA not only enhances the synthesis of triglyceride but also reinforces the phospholipid bilayer by double flipping event [16, 17]. OA ameliorates saturated FA-induced cell death by decrease of reactive oxygen stress (ROS) and ceramide. Moreover, exogenous OA treatment suppressed lipid ROS-induced ferroptosis [16, 18]. SCD catalyzes the biogenesis of MUFA from saturated fatty acid (SFA). Reversely, it has been shown that conjugation of OA with liver-X receptor increases the transcriptional expressions of SCD and sterol regulatory-element binding protein (SREBP) [19]. Loss of SCD results in aberrant lipid metabolism, leading to insulin resistance and lipotoxicity [20]. SCD deficiency also impairs mitochondrial function and affects β-oxidation, ATP production, and oxidative phosphorylation (OXPHOS) [21, 22]. In contrast to normal cells, cancer cells use of fatty acids to produce ATP for the rapid growth and actin polymerization [23, 24].

Although cancer stem cells (CSCs) account for a very small portion, they are responsible for tumor initiation, metastasis and tumor recurrence. Compared to the more differentiated cells that mostly rely on OXPHOS, CSCs have increased glycolysis as well as OXPHOS [25]. In CSCs, metabolic reprogramming from OXPHOS to glycolysis is facilitated by transcription factors including Sox2, Oct4 or Myc [26]. Additionally, lipid desaturation is crucial for the maintenance of cancer cell stemness [27]. Likewise, SCD is highly expressed in the CSC subpopulation of human bladder cancer [28], while silencing of SCD reduces CSC formation and mitigates cisplatin resistance [29].

The present study showed that lipogenesis is activated in CAFs via SCD upregulation under glucose-deficient conditions. And CAF-derived OA was transferred to cancer cells where they increased SCD expression and promoted stemness via nuclear translocation of yes-associated protein. Our data suggested that deregulated lipid metabolism and lipid desaturases could be a valuable target for the development of cancer therapy.

## Materials and methods

### Mouse xenograft models

Four five-week-old female BALB/c nude mice were purchased from Jungang Lab Animal, Inc. (Seoul, South Korea) and were subcutaneously injected with 1 × 10^7^ NCI-H460 (H460) cells suspended in matrigel (Corning, New York, USA). H460 cell line was purchased from Korea Cell Line Bank (KCLB Cat# 30,177, RRID:CVCL_UW76) and authenticated the source. All animal experiments were approved by the Institutional Animal Care and Use Committee of Seoul National University, Seoul, South Korea (SNU-190510–2-1). The resultant tumor masses from the four mice were resected for primary culture as described in the *Primary culture and fibroblast isolation* section. Parental, SCD-KO, and SCD-pCDH H460 cells were prepared as described in the *Generation of genetically engineered cells with dysregulated SCD expression* section. Mice were sacrificed four weeks after injection of the cells and the resultant tumor masses were collected.

### Cell culture and reagents

Complete medium contained 10 mM glucose with 10% fetal bovine serum (Invitrogen, Carlsbad, CA, USA), 10 mM HEPES, 2.0 g/l sodium bicarbonate, 0.3 g/l L-glutamine, 1 mM sodium pyruvate and 100 U per 100 μg ml − l penicillin/streptomycin (Sigma-Aldrich, St Louis, MO, USA). Glucose-deficient medium contained identical supplements with a complete RPMI medium except glucose. OA (Sigma-Aldrich) was dissolved in DMSO and cells were treated with 100 µM. SCD, lipid transporter, β-oxidation, and actin polymerization were chemically inhibited using CAY10566 (Cayman Chemical, Ann Arbor, MI, USA), sulfo-N-succinimidyl oleate (Cayman Chemical), etomoxir (Sigma-Aldrich), and latrunculin A (Cayman Chemical), respectively. Autophagy was blocked using CQ and 3-MA (Sigma-Aldrich).

### Primary culture and fibroblast isolation

Tissues containing underarm skin and xenografted tumor mass from four individual mice were used for primary culture. Primary cells passaged up to four times were used for further experiments. Fibroblasts were isolated from the primary cultured cells of tumor mass and skin tissues using a commercial kit (Miltenyi Biotec, Bergisch Gladbach, Germany) according to manufacturer’s instructions [30]. To confirm the phenotype of the isolated fibroblasts, flow cytometry was performed using FACSVerse (BD Biosciences, RRID:SCR_013311) with antibodies against FAP (AbCam, Cambridge, UK) and CD90 (Novus Biologicals, RRID:SCR_004286).

### Measurement of cell viability

Cell viability was measured using 3-(4,5-dimethylthiazol-2-yl)-2,5-diphenyl tetrazolium bromide assay (Sigma-Aldrich) [31]. The absorbance was measured at 570 nm using a microplate reader (BioTek Epoch, Izasa, Barcelona, Spain).

### Measurement of apoptosis

Apoptotic rate was measured using EzWay Annexin V-FITC apoptosis detection kits (Koma Biotech, Seoul, South Korea) as described in [31]. The cells analyzed with flow cytometry (BD Biosciences). Quadrant was divided according to control and percentage of annexin-V-positive cells were considered as rate of apoptotic cells.

### Analysis of fatty acid methyl esters

Harvested cell pellets and cell-free culture media were placed in tube with Teflon caps after freeze-drying. Methylating mixture [MeOH:Benzene:DMP (2,2-Dimethoxy-propane):H2SO4 = 39:20:5:2] and heptane were added to the tube and incubated at 80℃ for 2 h. After cooling the tubes at room temperature, two phases were observed. The upper phase containing fatty acid methyl esters (FAMEs) was extracted to conduct gas chromatography (Agilent Technologies, inc. Santa Clara, CA, USA) at NICEM of Seoul National University [32]. Quantitative validation of FAMEs was also performed at NICEM.

### Analysis of cell lipid droplets

Cultured cells were stained with BODIPY 493/503 (BODIPY, Invitrogen) according to manufacturer’s recommendation and analyzed by flow cytometry (BD Biosciences). Within BODIPY staining, P2 and P3 populations were established using untreated H460 cells. The staining of lipid droplets was assessed using a confocal laser scanning microscope (CLSM; Carl Zeiss, Göttingen, Germany).

### Nuclear fractionation

We isolated nuclear using Nuclear and Cytoplasmic Extraction kit (Invitrogen) according to manufacturer’s instructions. In brief, harvested cell pellets were resuspended with CER I buffer and CER II buffer. After centrifuge, cytoplasmic extract in the supernatant was transferred. Pellet was resuspended with NER buffer and centrifuged. Transfer nuclear extract was transferred and used for the experiment.

### Western blot assay

Membrane loaded with protein was incubated in blocking solution containing antibodies against SREBP-1 precursor and mature SREBP-1 (Santa Cruz biotechnology, CA, USA), SCD (Cell-Signaling technology, Danvers, MA, USA), mTOR (Cell-Signaling technology), BECN-1 (Santa Cruz biotechnology), LC3 (Novus Biologicals), ATG5 (Cell-Signaling technology), Oct4 (Santa Cruz biotechnology), Nanog (Cell-Signaling technology), YAP (Santa Cruz biotechnology), and β-actin (Cell-Signaling technology) overnight at 4 °C. Secondary horseradish peroxidase (HRP)-conjugated anti-rabbit (Santa Cruz biotechnology) or mouse (Santa Cruz biotechnology) antibody was added and incubated for 2 h. The protein expression was detected by chemiluminescence imaging system (ATTO) following the spread of Luminata Forte Western HRP Substrate (Merck Millipore). Densitometric analysis was performed using the ImageJ program (NIH, RRID:SCR_003070) (http://rsbweb.nih.gov/ij).

### Immunofluorescence staining assay

H460 cells were cultured in confocal dishes (SPL-life science, Pocheon, South Korea) and performed fixation, permeabilization and incubated with antibody. The cells were incubated with either Alexa Fluor 488 or 647-conjugated secondary antibody (Molecular Probes, Eugene, OR, USA). Nuclei were stained with DAPI contained mounting solution (Southern Biotechnology, Birmingham, AL, USA). Mitochondria in the H460 cells were stained using Mitotracker deep red (Invitrogen).

#### Measurement of free fatty acid (FFA)

The amount of FFA was measured using EZ-Free Fatty Acid Assay Kits (Dogen, Seoul, South Korea) according to manufacturer’s instruction. The absorbance was measured using a microplate reader (BioTek Epoch), and the amount of FFA was calculated based on the value of the standards.

### Quantitative real-time PCR

Total RNA was extracted using TRIzol reagent (Ambion, Austin, TX) and quantified by microplate reader (BioTek Epoch). Five hundred nanogram of total RNA was used for cDNA synthesis using TOPscript cDNA Synthesis kits (Enzynomics, Daejeon, Korea). Gene expression was quantified using a SYBR Green Real-time PCR kit (Enzynomics). Relative expression levels were normalized using GAPDH, and calculated using the ΔΔCt method [33]. Primer sequences are indicated in Additional file 2: Table S1.

### ATP measurement

ATP synthesis was assessed using the CellTiter-Glo Reagent (Promega, Madison, WI, USA) according to the manufacturer’s instructions. Briefly, detection reagent was added to each well and incubated at room temperature for 10 min. Subsequently, luminescence was measured using a luminometer (Infinite M200 Pro; Tecan, Mannedorf, Switzerland) after mixed thoroughly for 2 min.

### Measurement of mitochondrial membrane potential

Mitochondrial membrane potential was assessed by JC-1 assay (Invitrogen) according to the manufacturer’s instructions. The cells were incubated with 2 μM JC-1 solution at 37 °C for 30 min and analyzed using FACSVerse (BD Biosciences) after washing with PBS. As positive control, carbonyl cyanide 3-chlorophenylhydrazone (CCCP) was treated and used to evaluate the value of mitochondrial membrane potential.

### Measurement of mitochondrial calcium

To measure the mitochondrial calcium, the cells were stained with Rhod-2AM according to the manufacturer’s instructions. Harvested cells were stained with 2 μM Rhod-2AM for 30 min at 37 °C and analyzed the fluorescence by flow cytometry. The P2 population for Rhod-2AM staining was established using control.

### Flow cytometry

Harvested cells were incubated in blocking solution with primary antibodies against FAP (AbCam), CD90 (Novus Biologicals), CD133 (BD Biosciences), and CD44 (BD Biosciences) on ice. Cells were then further incubated with Alexa Fluor 488-conjugated anti-mouse secondary antibody (Molecular Probes) or Alexa Fluor 647-conjugated anti-rabbit secondary antibody (Molecular Probes) on ice and in the dark. Flow cytometry was performed using FACSVerse (BD Biosciences) immediately after washing with PBS.

### Autophagosome maturation detection

Autophagosome maturation was assessed using Cyto-ID autophagy detection kits (Enzo Life Sciences, Farmingdale, NY, USA) according to the instruction and analyzed by flow cytometry (BD bioscience). The level of autophagosome maturation in the cells was analyzed by comparing with the P2 population that was established using a negative control, untreated H460 cells.

### Spheroid formation

H460 cells were mixed with 2% B27 (Invitrogen), basic fibroblast growth factor (20 ng/mL) (Invitrogen), and epithelial growth factor (20 ng/mL) (Invitrogen) in complete medium or glucose-deficient conditions and placed in 6-well ultralow attachment plates (SPL life science) for 7 days. Over 100 μm of spheroids were determined by ImageJ software (NIH) and counted.

### Side population assay

To evaluate the percentage of side population, the cells were stained with Hoechst 33,342 (Sigma-Aldrich) as described in [34]. Briefly, 1 × 10^6^ cells were stained with 20 μg/mL Hoechst 33,342 for 90 min and 50 μM verapamil (Sigma-Aldrich) was treated to establish the population. After washing with PBS, we analyzed the population using flow cytometry at 355 UV light to detect the fluorescence with 450/20 band-pass filter and red fluorescence with a 675/20 filter.

### Generation of genetically engineered cells with dysregulated SCD expression

A human SCD-coding sequence with corresponding restriction sites, NheI and BamHI, was cloned and inserted into pCDH-EF1-MCS-T2A-puro lentiviral vector (BioCat Cat# CD500B-1 – CD523A-1, RRID:SCR_001440) to produce an SCD-pCDH construct. The pCDH-EF1 lentiviral vector was used as a vehicle control. The SCD-pCDH lentiviral vector was transfected to 293 T cells (ATCC Cat# CRL-3216, RRID:CVCL_0063) with second viral packaging reagents, and viral particles were collected. After incubation of H460 cells with the viral particles and polybrene (Merck Millipore), SCD-pCDH positive cells were selected by treatment with puromycin.

Either SCD-sgRNA-U6 plasmid (Additional file 2: Table S1) (Toolgen, Seoul, South Korea) or control-sgRNA was transfected into H460 cells with CRISPR/Cas9-puromycin-CMV plasmid vector (Toolgen) and lipofectamine-3000 (Invitrogen). Cell colonies that survived treatment with puromycin were individually cultured and followed by assessment of SCD expression by Western blot and RT-PCR. Subclones of the SCD-KO cells were used for further experiments Additional file 3: Table S1.

### Analysis of the correlation between SCD expression and survival in lung cancer patients

The data of correlation between SCD expression and prognosis in lung adenocarcinoma patients was acquired from Kaplan–Meier Plotter http://kmplot.com/analysis/index.php?p=service&cancer=lung [35, 36]. Hazard ratios, 95% confidence intervals, and log rank *P-*values were described for each result.

### Analysis of SCD expression in lung adenocarcinoma tissue microarray

IHC for SCD was performed in lung adenocarcinoma tissue microarray (US Biomax, Rockville, MD, USA) according to the manufacturer’s instructions [37]. The prepared tissues microarray slides were incubated with SCD antibody (Cell-Signaling technology) for overnight and incubated with horse anti-rabbit secondary antibody (Vector Laboratories, Burlingame, CA, USA) for 2 h. Following treatment with 3,3’-diaminobenzidine solution (Vector Laboratories), slides were counterstained using hematoxylin, dehydrated in alcohol, and mounted in a xylene-based solution. The mean IHC staining intensity for SCD was quantified using ImageJ software and scored 0–3 as follows [38]: 0, no staining; 1, < 15 mean intensity; 2, 15–50 mean intensity; and 3, > 50 mean intensity.

### Statistical analysis

In vitro experiments were performed independently at least three times and data values were presented as the mean ± SD. Statistical analyses were performed using GraphPad Prism Software (GraphPad Software, GraphPad Prism, RRID:SCR_002798). *P*-values were calculated by one-way analysis of variance or Tukey’s pairwise comparison. All results were considered statistically significant with *P*-values < 0.05.

## Results

### CAF culture supernatant boosted cancer cell malignancy under glucose-deficient conditions

**Fig. 1:**
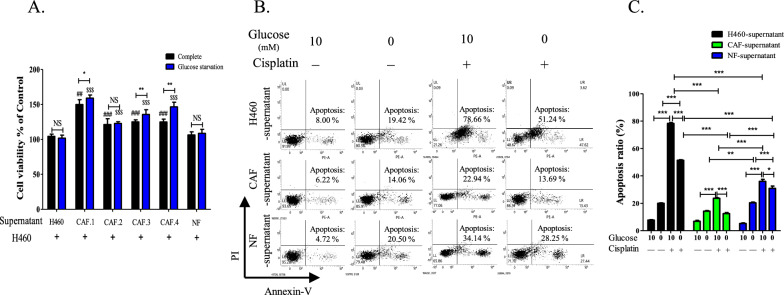
Viability and apoptosis were determined in the H460 cells treated with CAFs supernatant under glucose-deficient condition. **A** Viability of H460 cells cultured in complete medium or glucose-deficient conditions for one day followed by treatment with supernatant of H460, CAFs, or NF for one day. CAFs.1–4 was isolated from individual xenograft tumor mass. ^##^*P* < 0.01, ^###^*P* < 0.001 vs. H460 cells treated with H460 supernatant in complete medium. ^$$$^*P* < 0.001 vs. H460 cells treated with H460 supernatant in glucose-deficient conditions. ^**^P < 0.01, ^***^P < 0.001. Error bars indicate the standard error of the mean (SEM; n = 8). **B** Representative dot plot showing flow cytometry for apoptosis assay in H460 cells that were pretreated with supernatant of H460, CAF, or NF, followed by culture in complete or glucose-deficient medium containing cisplatin (10 μM) for one day. **C** Statistical analysis of apoptotic rate. Error bars indicate SEM (n = 3). ^*^P < 0.05, ^**^P < 0.01, and ^***^P < 0.001

CAFs and normal fibroblasts (NFs) isolated from primary cultures of four tumor masses and skin were validated by flow cytometry using antibodies against fibroblast activation protein (FAP) and CD90. In contrast to the negative staining in H460 cells, > 91% of CAFs and NFs were positive for FAP and CD90 (Additional file 1: Fig. S1A). To determine effects of cell-free media, supernatants were collected from the culture media from H460 cells, NFs, and CAFs. H460 cells were treated with the supernatants for 24 h, followed by incubation for further 24 h in either complete or glucose-deficient media. Compared to the NF and H460 supernatants, CAF-supernatant significantly enhanced the viability of H460 cells. Particularly, treatment with CAF 1, 3 and 4 supernatants increased the viability of H460 cells under glucose-deficient condition (Fig. 1A). Moreover, pretreatment with CAF supernatant for 24 h increased resistance to cisplatin under glucose-deficient conditions (Fig. 1B). Treatment with cisplatin for 16 h induced total apoptosis in H460 cells that were pretreated with H460 and NF supernatants by 79% and 34%, respectively. In contrast, the total apoptosis significantly decreased to 23% following pretreatment with CAF supernatant and was further suppressed to around 14% under glucose-deficient conditions. Although treatment with NF supernatant reduced total apoptosis compared with H460 supernatant under glucose-deficient conditions, it was lower than treatment with CAF supernatants (Fig. 1C). Under glucose-deficient condition, incubation of H460 cells with CAF supernatants exhibited a constant rate of apoptosis regardless of cisplatin treatment. Total apoptosis induced by cisplatin was lower in H460 cells treated with NF supernatant compared to the cells treated with H460 cell supernatant. This may be attributable to the growth factors secreted by fibroblasts (8).

### SCD upregulation in CAFs stimulated lipogenesis in cancer cells

**Fig. 2:**
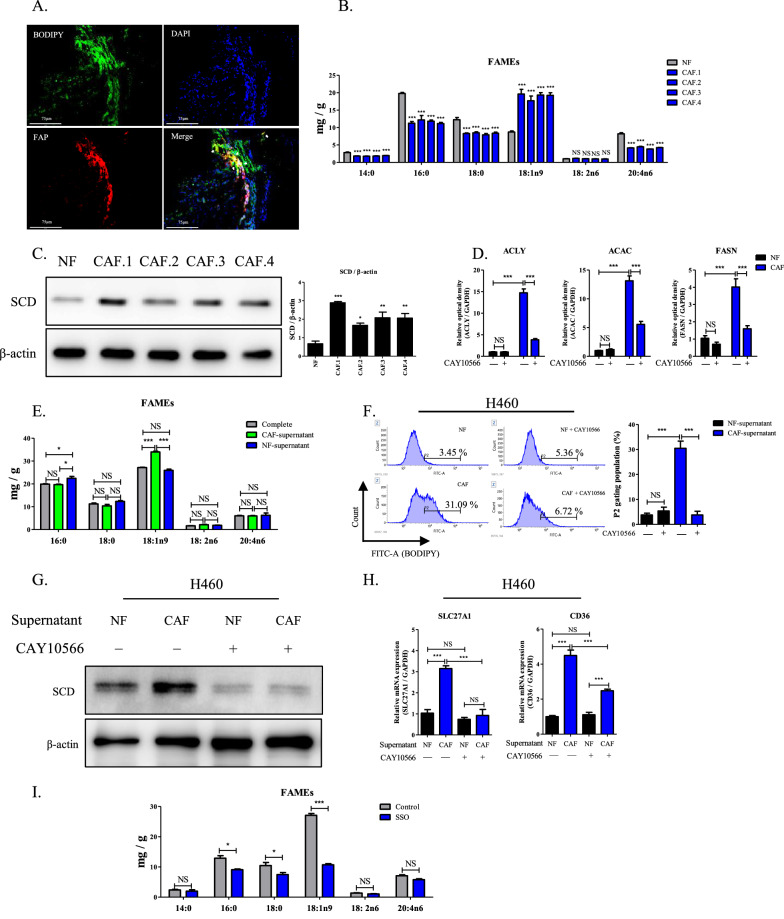
Transfer of CAF-derived lipids to cancer cells via upregulated lipid transporters. **A** Representative fluorescence image of FAP, BODIPY, and DAPI in xenografted tumor by transplanted in nude mice. 40 × magnification. Arrowhead indicates co-localized FAP and BODIPY fluorescence. Scale bar: 75 μm. **B** Profile of fatty acids content in cell pellets of primary cultured NF and CAF.1–4. ^***^P < 0.001, CAFs versus NF. Data represents the mean ± SEM (n = 3). **C** Expression of SCD and β-actin in NF and CAFs isolated from 1 to 4 individual tumor masses. ^*^P < 0.05, ^**^P < 0.01, and ^***^P < 0.001. Data indicate the mean ± SEM (n = 3). **D** Transcription levels of *ACLY, ACAC,* and *FASN* in NF and CAF.1 which cultured in complete medium and treated with CAY10566 (1 μm). ^***^P < 0.001. Data represent the mean ± SEM (n = 4). **E** Fatty acids profile in H460 cells treated with complete medium, NF and CAF.1 supernatant for 1 day. ^*^P < 0.05 and ^***^P < 0.001. Data represents the mean ± SEM (n = 3). **F** Representative histogram of BODIPY staining in H460 cells incubated in complete medium with supernatant of NF or CAF.1 for one day and subsequently treated with CAY10566. The bar graph indicated the mean value of P2 populations. Error bars represent SEM (n = 3). ^***^P < 0.001. **G** SCD protein expression and **H** mRNA expression of SLC27A1 and CD36 in H460 cells incubated in complete medium with supernatant of NF or CAF.1 for one day and subsequently treated with CAY10566. ^*^P < 0.05 and ^***^P < 0.001. Data represents mean ± SEM (n = 4). **I** Profile of fatty acid contents in H460 cells treated with 50 μM SSO and CAFs-supernatant for 1 day. ^*^P < 0.05 and ^***^P < 0.001. Data represents the mean ± SEM (n = 3)

In xenografted tumors, lipid levels were high in CAFs and gradually decreased away from the CAFs (Fig. 2A). Oil red O staining revealed a similar pattern of lipids, which was exclusively detected in CAFs in tumor masses (Additional file 1: Fig. S1B). Analysis of fatty acids (FAs) in cell pellets revealed that OA (C18:1n9) was higher than saturated fatty acids (SFA) involving palmitic acids (C16:0) and stearic acids (C18:0) in CAFs, but was lower in NFs (Fig. 2B). However, other MUFAs including linoleic acid (18:2n6) and arachidonic acid (20:4n6), were not higher in CAFs. Published data indicate that arachidonic acid-synthesized molecule is decreased in CAFs by miR-522 and promotes the invasion and growth of cancer cells [39]. Additionally, CAFs isolated from individual tumor mass showed higher lipids amount than NFs and H460 cells (Additional file 1: Fig. S1C). Notably, SCD expression was significantly higher in CAFs than NFs (Fig. 2C). To investigate the function of SCD in FA synthesis, CAFs and NFs were treated with CAY10566, a selective inhibitor of SCD, and mRNA levels of *ATP citrate lyase (ACLY), acetyl-CoA carboxylase (ACAC)*, and *fatty acid synthase (FASN)* were assessed. Comparative analyses revealed that high expression of SCD in CAFs increased the level of these genes but not in NFs (Fig. 2D). However, treatment of CAFs with CAY10566 reduced the amount of FA, particularly OA in the supernatant compared to non-treated counterpart (Additional file 1: Fig. S1D). Moreover, amount of saturated fatty acids was suppressed in supernatant from CAFs after treatment with CAY10566, suggesting the role of SCD in synthesis of fatty acid in CAFs. Accordingly, it has been shown that downregulation of lipogenesis genes in SCD-deleted mice suppresses both saturated fatty acids and monounsaturated fatty acids [40]. To determine whether CAF-derived FAs transferred to cancer cells, H460 cells were incubated with NF or CAF supernatant for one day, followed by measurement of FAs contents. OA was significantly higher in H460 cells treated with CAF supernatant than those treated with complete medium or NF supernatant, whereas content of SFAs was not changed by treatment with NF or CAF supernatants (Fig. 2E). The P2 population in H460 cells treated with NF supernatant was used as a comparison. Treatment of H460 cells with CAF supernatant significantly increased the P2 population to 31%, while it was reduced to 6.72% by CAY10566 treatment (Fig. 2F). In contrast, the P2 population in H460 treated with NF supernatant was not affected by CAY10566 treatment. To determine the role of FA from CAF-supernatant in H460 cells, we incubated H460 cells with supernatant from CAFs and NFs which were treated with CAY10566. In contrast to NF-supernatant, CAF supernatant increased SCD expression in H460 cells. However, CAFs supernatant from CAY10566-treated cells did not alter the SCD expression (Fig. 2G). Additionally, H460 cells treated with CAF supernatant showed increased expression of the lipid transporters, *CD36* and *SLC27A1* (Fig. 2H), but not *ACLY*, *ACAC*, and *FASN* (Additional file 1: Fig. S1E). Moreover, CAY10566 treatment significantly decreased the expression of *CD36* and *SLC27A1*. In contrast, treatment with NF supernatant did not affect the expression of lipid transporters in cancer cells. For analysis of fatty acid, H460 cells were incubated with CAF-supernatant and sulfo-N-succinimidyl oleate (SSO), an inhibitor of lipid transporter. Compared to the non-treated cells, the amount of OA was significantly lower in SSO-treated H460 cells (Fig. 2I), suggesting the OA was transferred from CAF-supernatant to H460 cells through lipid transporter. Moreover, treatment of H460 cells with OA enhanced the mRNA expressions of *SREBP-1c* and *FASN*. Confirming this finding, SSO treatment suppressed their expressions regardless of OA presence (Additional file 1: Fig. S1F).

### Lipid catabolism by cell-free FAs accelerated autophagy under glucose-deficient conditions

**Fig. 3:**
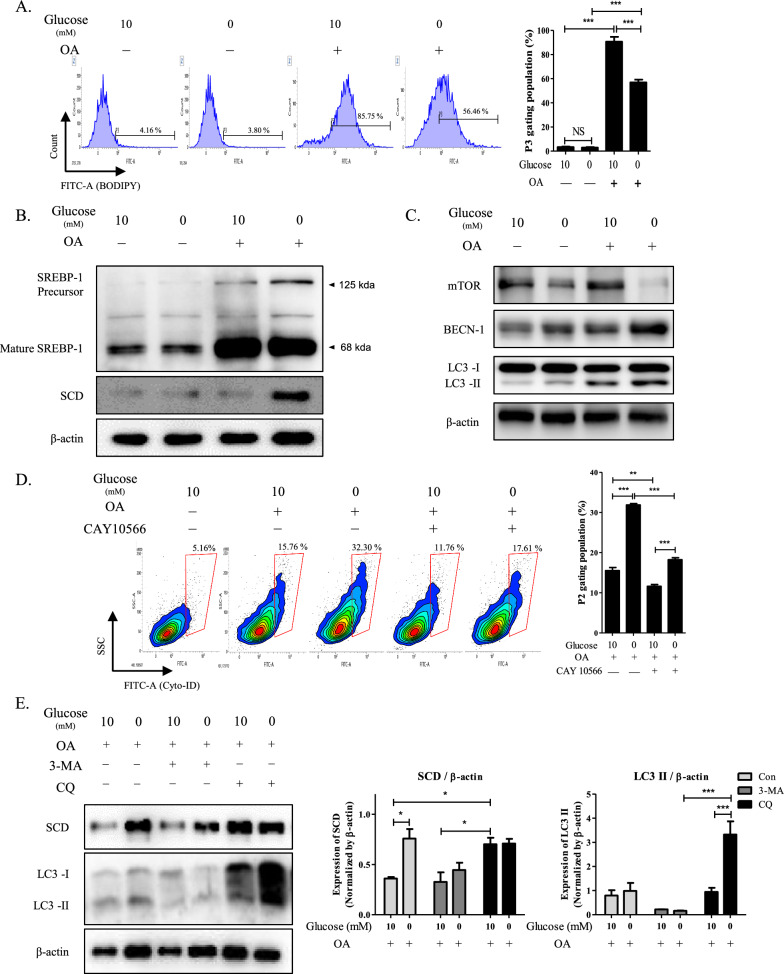
Increased SCD in glucose-starved H460 cells that were treated with OA enhances autophagic response. **A** Representative histogram showing BODIPY staining in H460 cells that were treated with OA in complete or glucose-deficient medium. The bar graph indicated the mean value of P3 populations. Error bars represent the SEM (n = 4). ^***^P < 0.001. **B** Protein expression of SREBP-1 precursor, mature SREBP-1, and SCD in H460 cells treated with OA in complete or glucose-deficient medium. ^*^P < 0.05, ^**^P < 0.01, and ^***^P < 0.001. Data indicate the mean ± SEM (n = 4). **C** Expression of mTOR, BECN-1, LC3-I, LC3-II, and β-actin in H460 cells treated with OA in complete or glucose-deficient medium. **D** Representative histogram of Cyto-ID staining of H460 cells treated with OA in complete or glucose-deficient medium. The bar graph indicated the mean value. ^***^P < 0.001. The results from each experiment represent the mean ± SEM (n = 3). **E** Expression of SCD, LC3-I, LC3-II and β-actin in H460 cells treated with OA and either 3-MA or CQ in complete or glucose-deficient medium. The bar graph indicated that the normalization of LC3-II and SCD expression by β-actin. Data represent the mean ± SEM (n = 3)

H460 cells treated with CAF supernatant showed greater fluorescence for lipids and FFA levels than those treated with NF supernatant and it was diminished under glucose-deficient conditions (Additional file 1: Fig. S2A, B). To further investigate of the role of cell-free FA, H460 culture media was supplemented with OA for one day. Lipid levels were evaluated in H460 cells using BODIPY staining. Addition of OA significantly increased the P3 population to approximately 85% in complete media, and to approximately 56% in glucose-deficient media (Fig. 3A). Expression levels of *SREBP-1c* and *SCD* were upregulated in H460 cells treated with CAF supernatant, which were further increased under glucose-deficient conditions (Additional file 1: Fig. S2C). Likewise, compared with cells cultured in complete media, lipid metabolism was further activated by OA treatment in glucose-starved cells, as indicated by upregulation of *SREBP*-1 precursor, *-1c,* and *SCD* (Fig. 3B, Additional file 1: Fig. S2D). In glucose-starved H460 cells, OA treatment increased the expression of autophagy indicators, BECN1 and LC3, but reduced mammalian target of rapamycin (mTOR) (Fig. 3C).

The P2 population of H460 cells were cultured in complete medium as a comparison to determine autophagosome using Cyto-ID. OA treatment increased the P2 population to 35%, which was further elevated to approximately 54% under glucose-deficient conditions (Additional file 1: Fig. S2E). Treatment with CAY10566 suppressed the P2 population by approximately 15% in glucose-starved H460 cells supplemented with OA (Fig. 3D). Additionally, under glucose-deficient conditions, OA treatment increased the expression of autophagosome markers, LC3-II and ATG5, which was reversed by treatment with CAY10566 (Additional file 1: Fig. S2F). Autophagosome maturation was targeted separately to assess the influence of autophagosome on SCD. Treatment with 3-methyladenine (3-MA) and chloroquine (CQ) blocked the autophagy response via inhibition of autophagosome formation and autophagosome–lysosome fusion, respectively [41]. OA treatment augmented the SCD and LC3-II expressions under glucose-deficient conditions compared to complete medium. However, 3-MA treatment suppressed their expressions in presence of OA and glucose deficiency (Fig. 3E). Moreover, supplement with CQ augmented SCD expression in complete medium compared to non-treatment.

### OA treatment augmented cancer cell stemness properties in glucose-deficient conditions

**Fig. 4:**
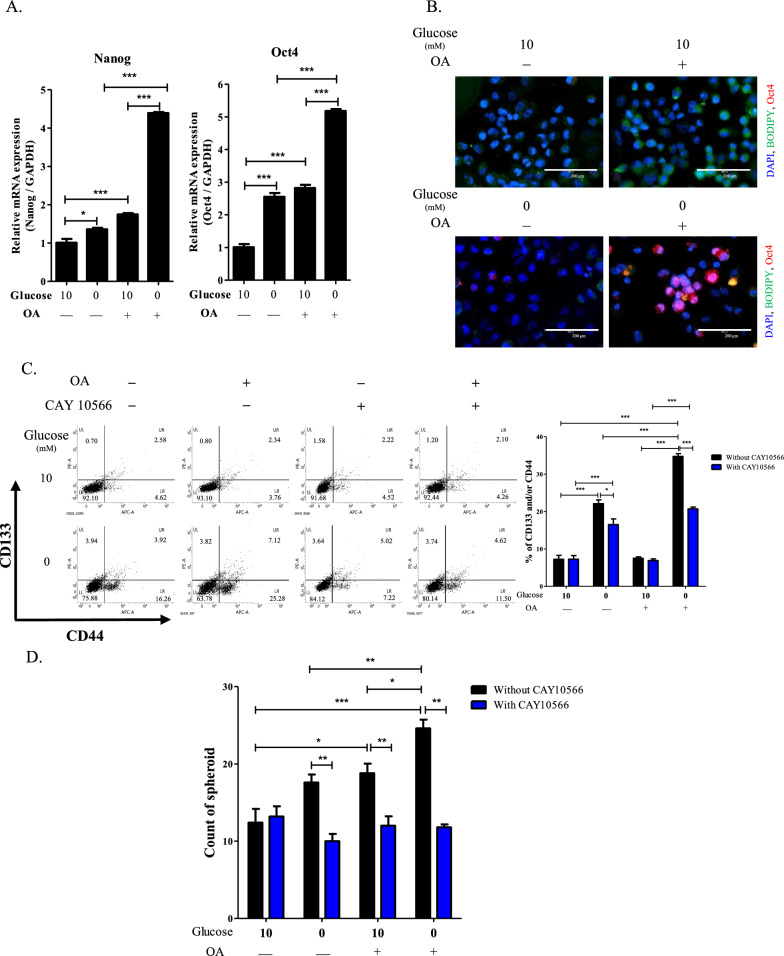
Effects of OA treatment on stemness of H460 cells under glucose-deficient condition. **A** Transcription levels of Nanog and Oct4 in H460 cells incubated with OA in complete or glucose-deficient medium for one day. ^*^P < 0.05 and ^***^P < 0.001. Data indicate the mean ± SEM (n = 4). **B** Representative fluorescence image of BODIPY and Oct4 in H460 cells cultured in complete medium or glucose-deficient conditions with/without OA treatment for 1 day. Green, BODIPY; red, Oct4; blue, DAPI. 20 × magnification. Scale bar: 200 μm. **C** Staining for CD133 and CD44 in H460 cells treated with OA and CAY10566 in complete or glucose-deficient medium for 1 day. ^*^P < 0.05 and ^***^P < 0.001. Bar graph indicates the percentage of CD133 and/or CD44 positive. Data represent the mean ± SEM (n = 3). **D** Number of spheroids derived from H460 cells treated with OA and CAY10566 in complete or glucose-deficient medium for 7 days. Spheroids over 100 μm in diameter were counted using Image J. ^*^P < 0.05, ^**^P < 0.01, and ^***^P < 0.001. Data represent the mean ± SEM (n = 5)

In H460 cells, expression of the stemness markers, *Nanog* and *Oct4*, were increased by OA treatment, which were further upregulated under glucose-deficient conditions (Fig. 4A). Lipid levels determined by BODIPY staining were enhanced when the presence of OA compared to its absence. However, glucose deficiency suppressed the BODIPY staining in the cells (Fig. 4B and Additional file 1: Fig. S2G). These results suggested that activated lipid metabolism under glucose-deficient conditions in the presence of OA may augment cancer stemness. Approximately 36.22% of H460 cells treated with OA were positive for the CSC markers, CD133 and CD44, under glucose-deficient conditions, and the population was reduced to 19.86% following CAY10566 treatment (Fig. 4C). Moreover, under glucose-deficient conditions, spheroid formation in H460 cells was enhanced by OA treatment, and was reversed by CAY10566 treatment (Fig. 4D). Additionally, CAY10566 treatment induced a consistent the number of spheroids under glucose deficient conditions regardless of OA treatment.

### *SCD-enriched cancer stemness was achieved *via* F-actin-mediated YAP nuclear translocation*

**Fig. 5:**
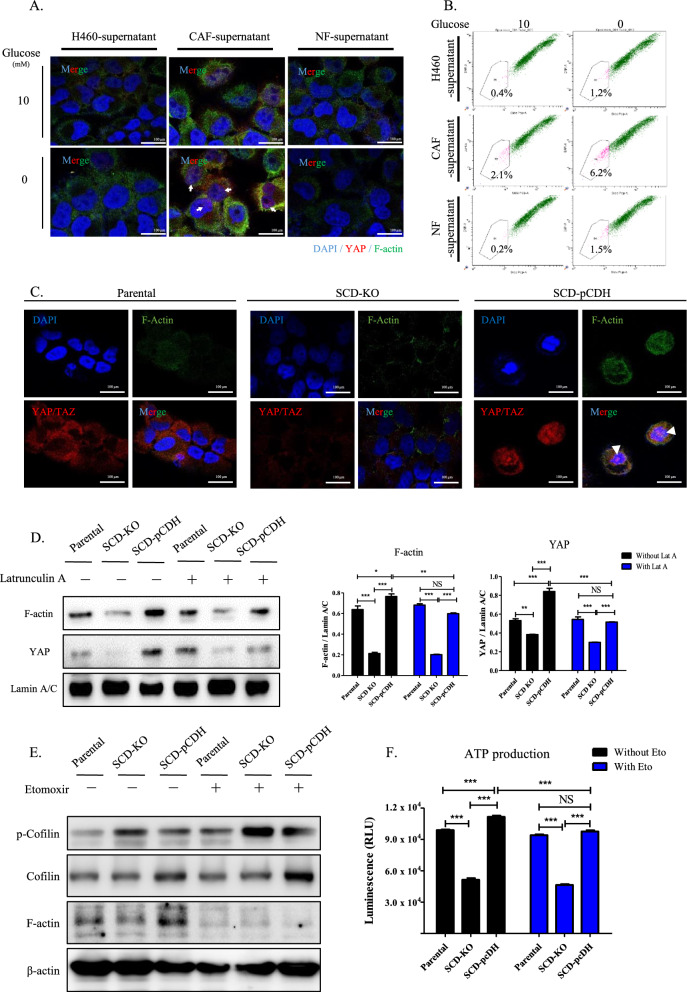
Increased SCD promotes stemness via translocation of YAP into the nucleus. **A** Representative fluorescence image of YAP, F-actin and DAPI in H460 cells cultured with supernatant of H460, NF or CAF in complete or glucose-deficient medium for one day. 40 × magnification. Arrowhead indicates co-localization of YAP and DAPI. Scale bar: 100 μm. **B** Representative dot plot of flow cytometry for side population assay. H460 cells incubated with supernatant of H460, NF or CAF followed by further culture in complete or glucose-deficient medium for one day. The P6 population was established using verapamil treatment (negative control). **C** Representative fluorescence image of YAP, F-actin and DAPI in parental, SCD-KO and SCD-pCDH H460 cells. Arrowhead indicates co-localized fluorescence of YAP and F-actin in the nucleus. 40 × magnification. Scale bar: 100 μm. **D** Expression of F-actin, YAP and lamin A/C in the nucleus of parental, SCD-KO and SCD-pCDH H460 cells treated with Lat A and normalized using lamin A/C using ImageJ. ^*^P < 0.05, ^**^P < 0.01, and ^***^P < 0.001. Data represent the mean ± SEM (n = 3). **E** Expression of p-cofilin, F-actin, and β-actin and (F) level of ATP in parental, SCD-KO, and SCD-pCDH H460 cells treated with etomoxir. ^***^P < 0.001. Data indicate the mean ± SEM (n = 9)

Under glucose-deficient conditions, yes-associated protein (YAP) and F-actin were mainly detected in the nuclei of H460 cells treated with CAF supernatant (Fig. 5A). In contrast, only faint YAP fluorescence was detected in the cytoplasm of H460 cells treated with either H460 or NF supernatant, which was not affected by glucose starvation. The abundance of side populations (SPs) in H460 cells was increased by treatment with CAF supernatant, which was further enhanced by glucose starvation, but not in cells treated with H460 or NF supernatant (Fig. 5B).

Genetically-engineered subclones of H460 cells were manufactured by lentiviral vector or CRISPR/cas9-based gene editing to elucidate the mechanisms involved in the control of cancer stemness by SCD. Compared with parental and mock cells, expression of *SCD* mRNA and protein were completely abrogated in SCD-KO cells (Additional file 1: Fig. S3A, B). SCD-pCDH cells had overexpressed SCD, while no change of SCD was observed in control Cas9 and pCDH transfected cells (Additional file 1: Fig. S3C). Additionally, analysis of FAs revealed that OA content was higher in SCD-pCDH cells than other subclones (Additional file 1: Fig. S3D). Downregulation of SCD decreases the MUFA content, but increases the content of SFA and lactate level [42]. Upregulated lactate by SCD inhibition might produce the content of SFA [43]. Moreover, the expressions of autophagy indicators, BECN1 and Atg5, were increased in SCD-pCDH cells but suppressed in SCD-KO cells compared to parental cells (Additional file 1: Fig. S3E).

Higher expression levels of Nanog and Oct4 were observed in SCD-pCDH cells compared with the parental cells; however, their expression levels were significantly lower in SCD-KO cells (Additional file 1: Fig. S3F). While only 7.61% of SCD-KO cells were positive for CD133 and/or CD44, 45% of SCD-pCDH cells were positive for these markers (Additional file 1: Fig. S3G). SP analyses in parental, SCD-KO, and SCD-pCDH cells revealed that the abundance of SPs was positively associated with SCD expression (Additional file 1: Fig. S3H). The number of spheroids was higher in SCD-pCDH cells than parental and SCD-KO cells. Additionally, OA treatment increased the spheroids in parental and SCD-pCDH cells, while the number of spheroid in SCD-KO cells was not affected by OA treatment (Additional file 1: Fig. S3I).

In contrast to the cytoplasmic localization in parental cells, high levels of YAP and F-actin were detected in the nuclei of SCD-pCDH cells (Fig. 5C, Additional file 1: S3J). SCD-KO cells showed lower expression of YAP and F-actin in the nuclei and cytoplasm compared with the parental cells. Cells were treated with an F-actin inhibitor, latrunculin A (Lat A), to evaluate the role of F-actin in the nuclear translocation of YAP. Nuclear was isolated from whole lysate and assessed their expressions (Additional file 1: Fig. S3K). Lat A treatment significantly suppressed nuclear expression of YAP and F-actin in SCD-pCDH cells (Fig. 5D), but not in parental or SCD-KO cells.

Strong fluorescence for F-actin levels were detected in the nuclei, with elongation of mitochondrial dynamics in SCD-pCDH cells (Additional file 1: Fig. S4A), whereas mitochondrial fragmentation was observed in SCD-KO cells. Moreover, analyses of mitochondrial membrane potential and mitochondrial calcium in parental, SCD-KO, and SCD-pCDH cells revealed that SCD played a crucial role in the integrity of mitochondrial function (Additional file 1: Fig. S4B, C). In mitochondria, activation of β-oxidation increases the ATP production by upregulation of the biogenesis. Moreover, ATP is necessary to reinforce the actin polymerization by stabilization the capping [44]. To determine actin polymerization was regulated by SCD-mediated β-oxidation, subclones of H460 cells were treated with the β-oxidation inhibitor, etomoxir (Eto). In SCD-pCDH cells, treatment with Eto suppressed ATP production and expression of F-actin, but increased p-cofilin (Fig. 5E, F). The level of ATP was lower in SCD-KO cells than parental cells, and were not affected by Eto treatment in these subclones. Additionally, Eto treatment suppressed the translocation of YAP in nuclei of SCD-pCDH cells (Additional file 1: Fig. S4D).

### SCD promoted tumorigenesis in a mouse xenograft model

**Fig. 6:**
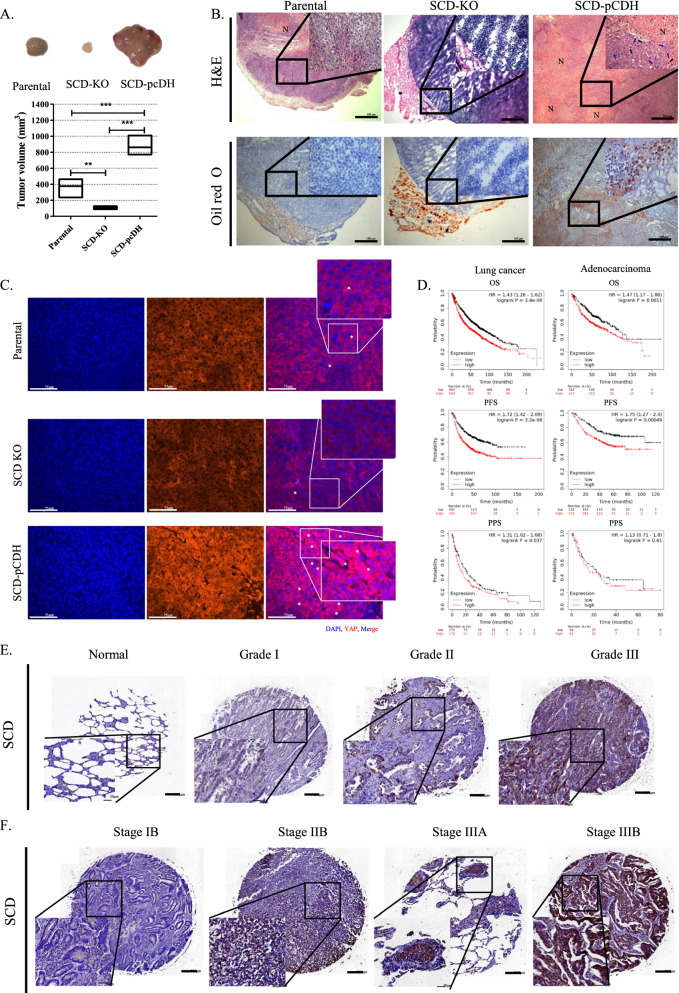
SCD promotes tumorigenesis with nuclear localization of YAP. **A** Size of xenografted tumor following injection of parental, SCD-KO, and SCD-pCDH H460 cells. ^**^P < 0.01 and ^***^P < 0.001. Data indicate the mean ± SEM (n = 3). **B** Representative image of hematoxylin and eosin and oil red O staining in xenografted tumors from parental, SCD-KO, and SCD-pCDH H460 cells. 4 × and 40 × magnification. Black and blue arrows indicate mitotic cell and multi nucleolus, respectively. N: necrosis region. **C** Representative fluorescence image of YAP and DAPI staining in xenografted tumors from parental, SCD-KO, and SCD-pCDH H460 cells. 20 × magnification. Scale bar: 75 μm. Asterisk indicates co-localized YAP and DAPI. **D** Analysis of survival rate (OS, PFS and PPS) in patients with lung cancer (n = 1925, 982, and 344, respectively) or lung adenocarcinoma (n = 719, 461, and 125, respectively) based on SCD expression using Kaplan–Meier plot of follow-up for 200 months. The level of SCD expression is divided with a median cutoff value. **E** Representative IHC staining for SCD in normal lung tissue and lung adenocarcinoma tissue microarray. 5 × and 20 × magnification. **F** Representative IHC staining for SCD in stage IA, IIA, IIB, and IIIA lung adenocarcinoma tissue microarray. 5 × and 20 × magnification

Parental, SCD-KO, or SCD-pCDH subclones of H460 cells were subcutaneously injected into immunodeficient mice, respectively. After four weeks, the tumor size was significantly larger in mice injected with SCD-pCDH cells than those with other subclones (Fig. 6A). Furthermore, tumors generated from SCD-KO cells were smaller than those from parental cells. Histologically, a large amount of lipids was detected not only within cancer cells, but also in the extracellular space of the tumor tissues generated from SCD-pCDH cells, whereas lipids were not observed in the tumor tissues from parental or SCD-KO cells (Fig. 6B). The SCD expression was higher in xenografted tumors produced by SCD-PCDH cells, than in those from parental cells, whereas its expression was not detected in tumors from SCD-KO cells (Additional file 1: Fig. S5A). Additionally, high level of lipids was also detected in the stromal cells. Additionally, aggressive features of cancer cells were observed in the SCD-pCDH subclone-derived tumor, as characterized by numerous mitotic figures, multiple large nucleoli, and necrosis (Fig. 6B). Tumor tissue generated from parental cells showed tumor cells arranged in solid sheet and glandular structures. In contrast, tumors derived from the SCD-KO subclone were encapsulated and had diffuse glandular structures. YAP fluorescence was faintly detected in the cytoplasm of cancer cells in parental or SCD-KO cell-derived tumors, whereas high levels of fluorescence were detected in the nuclei of cancer cells in SCD-pCDH cell-derived tumors (Fig. 6C). The YAP staining in the nucleus was higher in SCD-pCDH cell-derived tumors than that in parental and SCD-KO cells (Additional file 1: Fig. S5B).

To further assess the value of SCD expression as a prognostic biomarker of survival rate, Kaplan–Meier plots were implemented for patients with lung adenocarcinoma with follow-up analysis for 200 months (http://kmplot.com/analysis/index.php?p=service&cancer=lung). The analysis revealed that 1925 lung cancer and 719 adenocarcinoma patients with low expression of SCD had longer overall survival (OS), progression-free survival (PFS), and post-progression survival (PPS) than the patients with high expression of SCD (Fig. 6D). Specifically, the median PFS in lung cancer and adenocarcinoma patients with low SCD expression was 27.14 and 41.4 months, respectively, and was 12.1 and 18.63 months, respectively, in patients with high SCD expression. The Cancer Genome Atlas (TCGA) data [45] revealed that the lung adenocarcinoma patients with high SCD expression had lower survival rate than the patients with low SCD expression (Additional file 1: Fig. S5C). Likewise, Immunohistochemistry (IHC) for SCD expression on human lung tissue array revealed that the mean intensity score of SCD staining was higher in adenocarcinoma compared with normal lung tissue, and was positively associated with increase of tumor grade and clinical stage (Fig. 6E, F). Indeed, the percentage of SCD positive cells was increased with the grade and stage in lung adenocarcinoma tissues (Additional file 1: Fig. S5D).

### Discussion

Functional changes of non-cancerous stromal cells influence cancer cell malignancy. CAFs are the main component of the tumor stroma and interact with cancer cells via direct or indirect crosstalk within the tumor microenvironment [46]. Mediators released from the CAFs contribute to cancer cell growth and chemoresistance [11, 47, 48]. The present study revealed that CAFs significantly increased the synthesis of OA that was transferred to neighboring cancer cells. Moreover, the CAF-derived OA enhanced cancer cell stemness via upregulation of SCD and downstream signaling pathways in In vitro and In vivo models.

Compared with NFs, lipid metabolism is significantly enhanced in CAFs [2, 49]. Our study also showed that the lipid metabolism was reprogrammed to increase the OA in CAFs under glucose deficient condition through activation of SCD. Furthermore, we found that the OA released from the CAFs was transferred to the lung adenocarcinoma cells through the lipid transporters. Analogously, our xenograft model revealed that lipid was predominantly detected in CAFs and its concentration was higher in the areas near CAFs. Our data indicated that OA derived from CAFs contributed to the reprogramming of lipid metabolism in cancer cells.

Unlike normal tissue, tumor growth requires a massive supply of glucose and oxygen for ATP production, resulting in glucose-deficient conditions [11, 50]. In turn, glucose deficiency modifies mitochondrial activity that affects the cancer cell phenotype [51]. Moreover, glucose starvation attenuates the response to cisplatin-based chemotherapy by upregulation of AMPK [52, 53]. Under glucose-deficient condition, lipid metabolism is activated by the autophagic response in cancer cells, which functions as an alternative energy source [54]. We demonstrated that autophagy and lipid metabolism were highly activated in lung adenocarcinoma cells by OA-transferred from CAFs under glucose-deficient condition. Additionally, we showed that SCD is essential for autophagosome maturation, particularly in the fusion with lysosomes. In line with our results, previous study reported that silencing of SCD interferes the fusion of autophagosome and lysosome [55]. Furthermore, we revealed that autophagosome synthesis regulated the SCD expression in cancer cells under the glucose deficient condition. The importance and detailed mechanisms involved in SCD regulation by autophagosome are limited and warrant further investigation.

Growing body of evidence indicates that CSCs exbibit a self-renewal capacity and express stemness markers like OCT4, NANOG and SOX2. And their stemness properties are maintained by intracellular mechanisms such as Wnt/β-catenin and Hippo pathways. Recent studies have shown that increased expression of SCD is correlated with cancer malignancy and promotes cancer stemness [27, 56]. It was shown that the chemical inhibitor of SCD suppressed tumor growth in mouse model [57]. In lung adenocarcinoma, an effector of Hippo pathway, YAP, plays a crucial role in the maintenance of stemness by physical interaction with Sox2 and Oct4 [58]. Nuclear translocation of YAP is essential for the maintenance of stemness, which is mediated by F-actin polymerization [59]. Expression of F-actin is significantly suppressed by SCD-inhibitor in lung adenocarcinoma [60]. Thus far, the molecular mechanism involved in SCD regulation of F-actin formation and nuclear YAP translocation was not comprehensively understood. Our study demonstrated that ATP production by β-oxidation was significantly increased in SCD-overexpressing cancer cells and was provided as a fuel for F-actin polymerization and nuclear translocation of YAP. Moreover, our xenograft model using cancer cell subclones with manipulated SCD expression also supported the role of SCD in the maintenance of CSC properties. The SCD-overexpressing subclones produced larger tumor masses that comprised of more malignant cells with higher level of nuclear YAP than those from the parental or KD subclones. Our data indicated that activation of the lipid metabolism by SCD is critical for the maintenance of stemness of cancer cells under glucose deficient condition.

The present study also explored the clinical relevance of SCD expression in lung cancer patients. Our data indicated that SCD expression was negatively correlated with survival rate in patients with lung adenocarcinomas. Moreover, SCD expression levels were increased along with tumor grade and stage, but was not detected in normal lung tissue. Previous reports showed that SCD expression was associated with stemness markers, such as CD24, CD133, SOX2, and CD44 [29, 61]. Collectively, our data indicate that increased level of SCD is associated with aggressiveness and poor prognosis of lung adenocarcinoma.

Taken together, our study illustrates that high level of OA in CAF-supernatant is transferred to lung adenocarcinoma cells via lipid transporter and reprograms the lipid metabolism by upregulated SCD under glucose-deficient conditions. Furthermore, the increased SCD expression augments stemness of cancer cell through actin-polymerization and subsequent YAP nuclear translocation. Clinically, high level of SCD expression is associated with poor prognosis in lung cancer patients. These findings suggest that regulation of the lipid metabolism by targeting SCD could be a potential way to develop clinically relevant therapies.

## Supplementary Information

**Additional file 1. Figure S1.** Phenotype of CAFs isolated from xenografted tumor masses (A) Staining for CD90 and FAP in H460 cells, NFs and CAFs. (B) Representative image of oil red O staining in a xenografted tumor. Red fluorescence indicates oil red O staining. 4× magnification. Scale bar: 150 μm. (C) Representative histogram for BODIPY staining in H460 cells, CAFs and NFs. The P2 population was established using H460 cells. ***P < 0.001 vs. H460 cells. Data indicate the mean ± SEM (n = 3). (D) Profile of fatty acid contents in supernatant from control and 1 μM CAY10566-treated CAFs. *P < 0.05, **P < 0.01, and ***P < 0.001. Data represent the mean ± SEM (n = 3). (E) Transcription levels of ACLY, ACAC, and FASN in H460 cells incubated in complete medium with supernatant of NF or CAF.1 for one day and subsequently treated with CAY10566. *P < 0.05. Data represent the mean ± SEM (n = 4). **Figure S2.** Effect of glucose deficiency on lipid metabolism and autophagosome. (A) Representative fluorescence image of BODIPY staining in H460 cells incubated with NF or CAF supernatant in complete or glucose-deficient medium for one day. 20× magnification. Scale bar: 50 μm. (B) Assessment of FFA levels in H460 cells incubated with supernatant of NF or CAF in complete or glucose-deficient medium for one day. $$P < 0.01, $$$P < 0.0001 vs. treatment of H460 cells with H460 supernatant. #P < 0.05, ###P < 0.0001 vs. treatment of glucose starved H460 cells with H460 supernatant. **P < 0.01 and ***P < 0.001. Error bars indicate SEM (n = 4). (C) Transcription levels of SREBP-1c and SCD in H460 cells incubated with supernatant of NF or CAF and cultured in complete or glucose-deficient medium for one day. #P < 0.05, ^##^P < 0.01, ^###^P < 0.001 vs. treatment of H460 cells with H460 supernatant. ^$$^P < 0.01, ^$$$^P < 0.001 vs. treatment of glucose starved H460 cells with H460 supernatant. *P < 0.05, **P < 0.01, and ***P < 0.001. Error bars indicate SEM (n = 3). (D) Transcription levels of SREBP-1c and SCD in H460 cells treated with OA in complete or glucose-deficient medium. *P < 0.05, **P < 0.01, and ***P < 0.001. Data indicate the mean ± SEM (n = 4). (E) Representative flow cytometric analysis of Cyto-ID staining in H460 cells treated with OA and CAY10566. The bar graph indicated the mean value. **P < 0.01 and ***P < 0.001. Data represent the mean ± SEM (n = 3). (F) Expression of ATG5, LC3-I, LC3-II, and β-actin in H460 cells treated with OA in complete or glucose-deficient medium. (G) Representative fluorescence image of Oct4, BODPY, and DAPI in H460 cells treatment with OA or not in the presence or absence of glucose. 20× magnification. Scale bar: 200 μm. **Figure S3.** Characterization of SCD gene-manipulated cells and their side population (SP) (A) Protein expression and (B) transcription level of SCD in control Cas9 (Mock) cells and colony of SCD-KO cells. **P < 0.01 vs. mock cells. Data indicate the mean ± SEM (n = 4). (C) Expression of SCD in parental, control Cas9, SCD-KO, control-pCDH, and SCD-pCDH H460 cells. (D) Profile of fatty acid contents in subclones of H460 cells. *P < 0.05, **P < 0.01, and ***P < 0.001, Data represents the mean ± SEM (n = 3). (E) Expressions of Atg5 and BECN1 in parental, SCD-KO, and SCD-pCDH H460 cells. (F) Expression of Nanog, Oct4 and β-actin, and (G) staining for CD133 and CD44 in parental, SCD-KO, and SCD-pCDH H460 cells. (H) Representative flow cytometry for SP assay in H460 cells treated with verapamil, parental, SCD-KO, and SCD-pCDH H460 cells. ***P < 0.001. Data indicate the mean ± SEM (n = 3). (I) Representative image for spheroid assay in SCD subclones in the presence or absence of OA. Spheroids over 100 μm in diameter were counted using Image J. *P < 0.05, **P < 0.01, and ***P < 0.001. Data represent the mean ± SEM (n = 5). (J) Normalization of phosphor-cofilin and F-actin with cofilin and β-actin. Data represent the mean ± SEM (n = 3). (K) Expression of β-actin, α-tubulin, and Lamin A/C in whole lysate, cytoplasmic, and nuclear of H460 cells. **Figure S4.** Effects of SCD on mitochondrial function and dynamics machinery. (A) Representative fluorescence image of mitotracker, F-actin, and DAPI in parental, SCD-KO, and SCD-pCDH H460 cells. Arrow indicates co-localized fluorescence of F-actin and mitotracker. 40× magnification. Scale bar: 100 μm. (B) Representative flow cytometry for JC-1 staining in parental, SCD-KO, and SCD-pCDH H460 cells. CCCP was used as a positive control to determine the proportion of red and green regions. ***P < 0.001. Data indicate the mean ± SEM (n = 3). (C) Representative flow cytometry of Rhod-2AM staining in parental, SCD-KO, and SCD-pCDH H460 cells. The P2 population was established using parental H460 cells. ***P < 0.001. Data represents the mean ± SEM (n = 3). (D) Representative fluorescence image of YAP and DAPI in parental and SCD-pCDH cells in presence or absence of etomoxir. 40× magnification. Scale bar: 10 μm. **Figure S5.** Association of SCD expression in tissues of lung cancer patients (A) Representative IHC staining for SCD in xenografted tumors generated from parental, SCD-KO, and SCD-pCDH cells. 5× and 20× magnification. (B) The mean fluorescence of DAPI and YAP co-localization was measured using ImageJ software. **P < 0.01, and *** P < 0.001. Data indicate the mean ± SEM. (C) Overall survival rates of low- and high-SCD groups in lung adenocarcinoma patients that were available in TCGA datasets. (D) Statistical analysis of DAB intensity is based on quantification of the percentage of SCD+ area in each TMA tissue core using ImageJ software. *P < 0.05, **P < 0.01, and *** P < 0.001. Data indicate the mean ± SEM.

**Additional file 2.** **Table S1. **SCD primer sequences and single guide RNA.

**Additional file 3.** **Table S2.** Normalized FAMEs data and list of delta-delta Ct values for qPCR.

## Data Availability

The data in this manuscript are available when request to the corresponding author.
