# Supplementary material for: Oleic acid from cancer-associated fibroblast promotes cancer cell stemness by stearoyl-CoA desaturase under glucose-deficient condition
Source: Cancer Cell Int. 2022 Dec 13;22:404. doi: 10.1186/s12935-022-02824-3 (PMC9746202; doi:10.1186/s12935-022-02824-3)

Additional Figure. S1

Additional

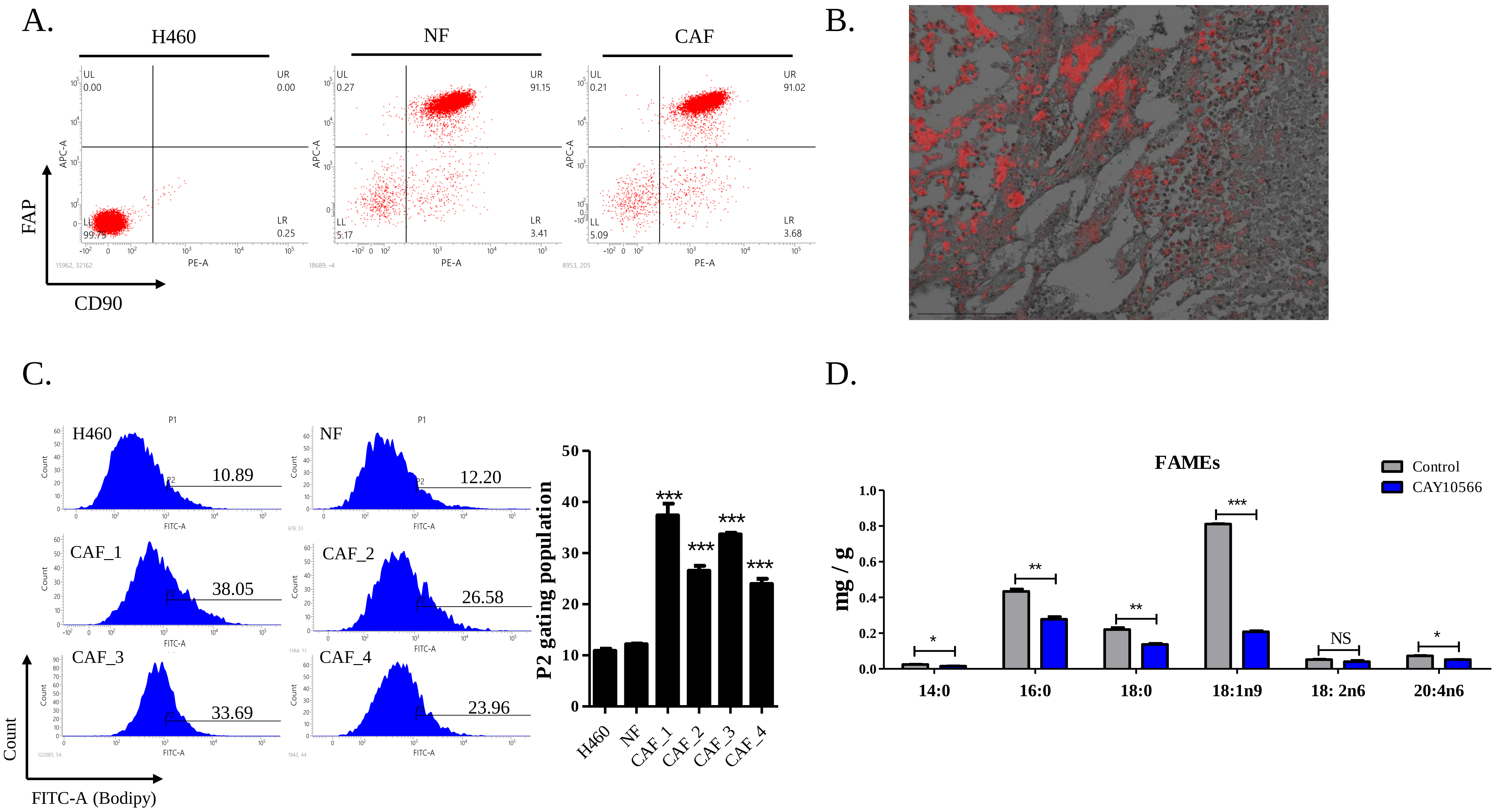

Additional Figure. S1

Additional

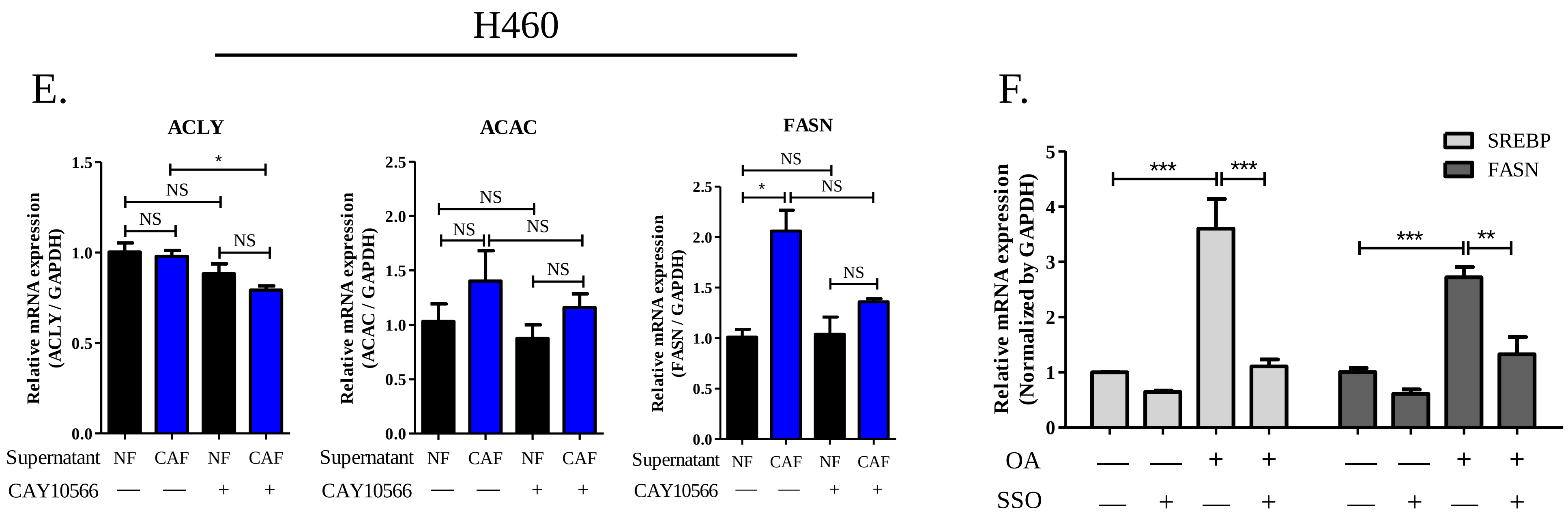

Additional Figure. S2

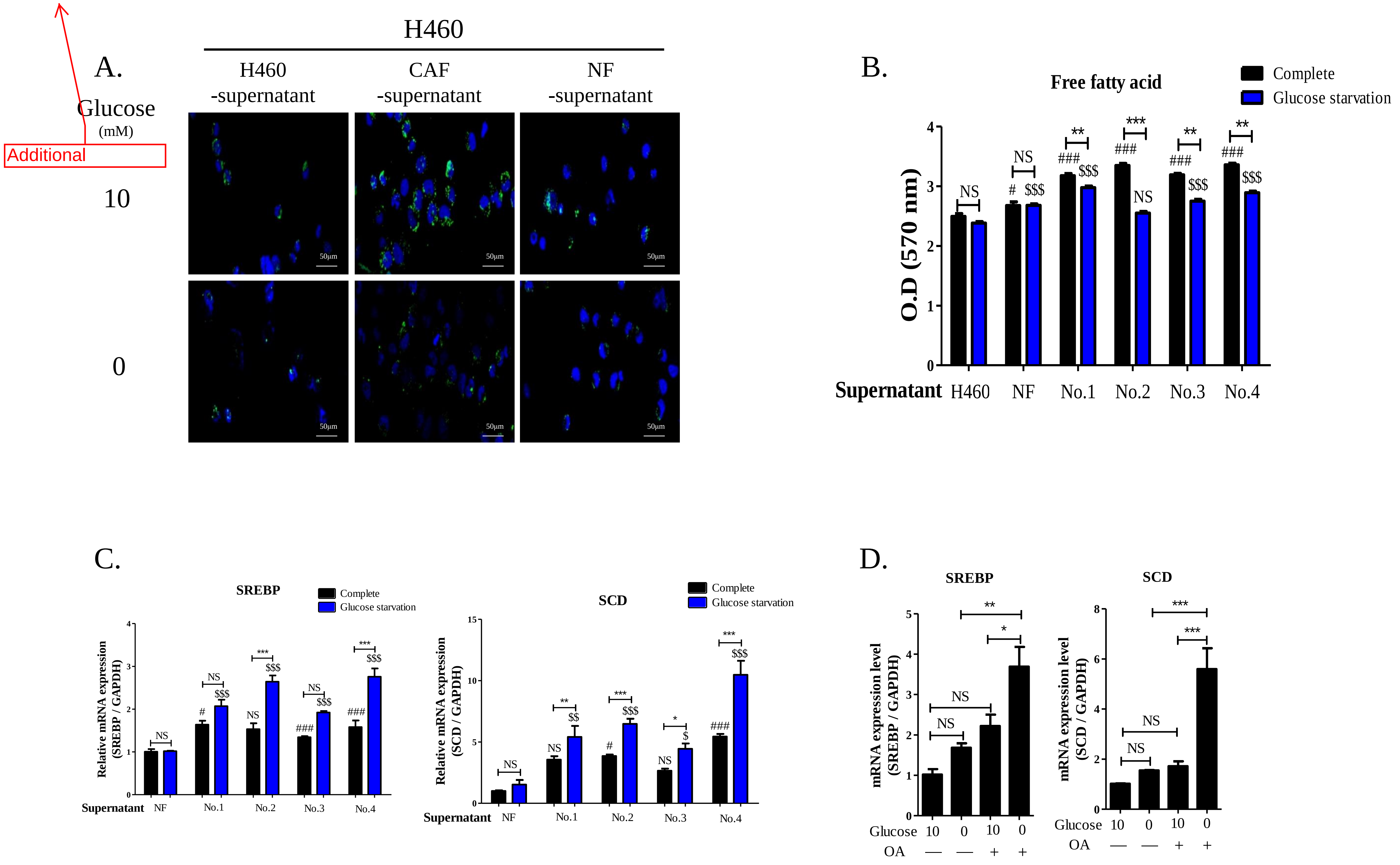

Additional Figure. S2

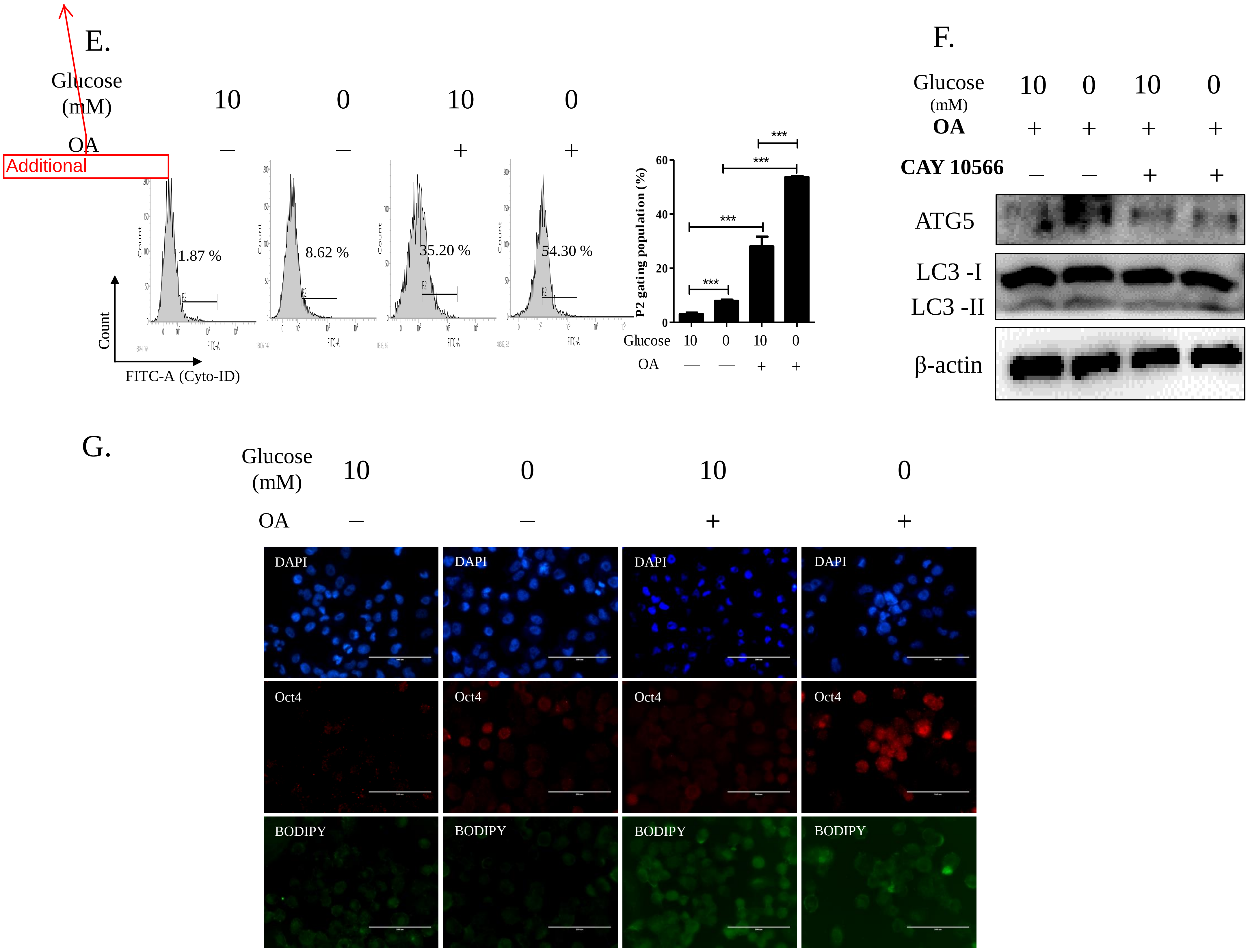

Additional Figure. S3

Additional

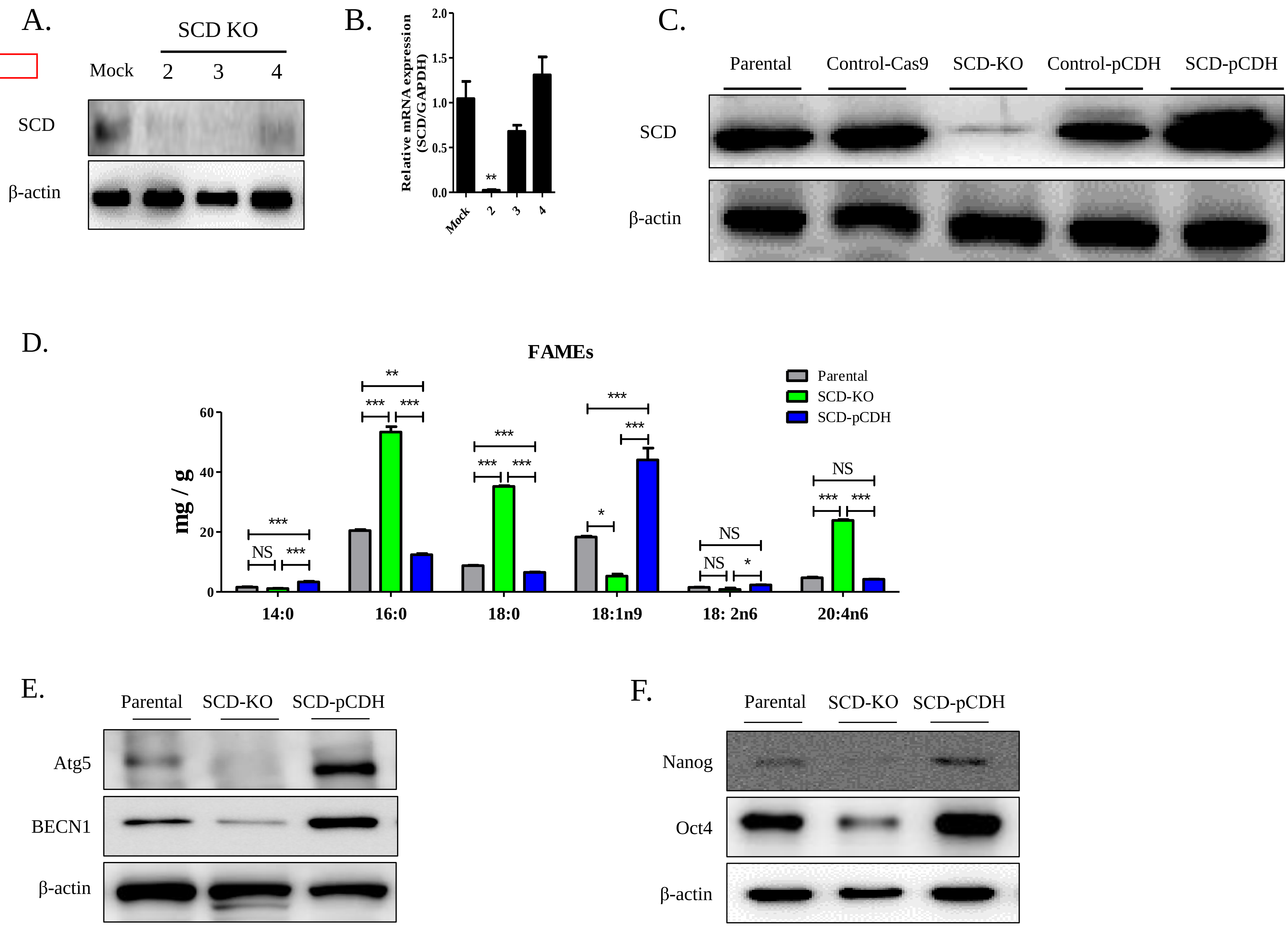

Additional Figure. S3

Additional

G.

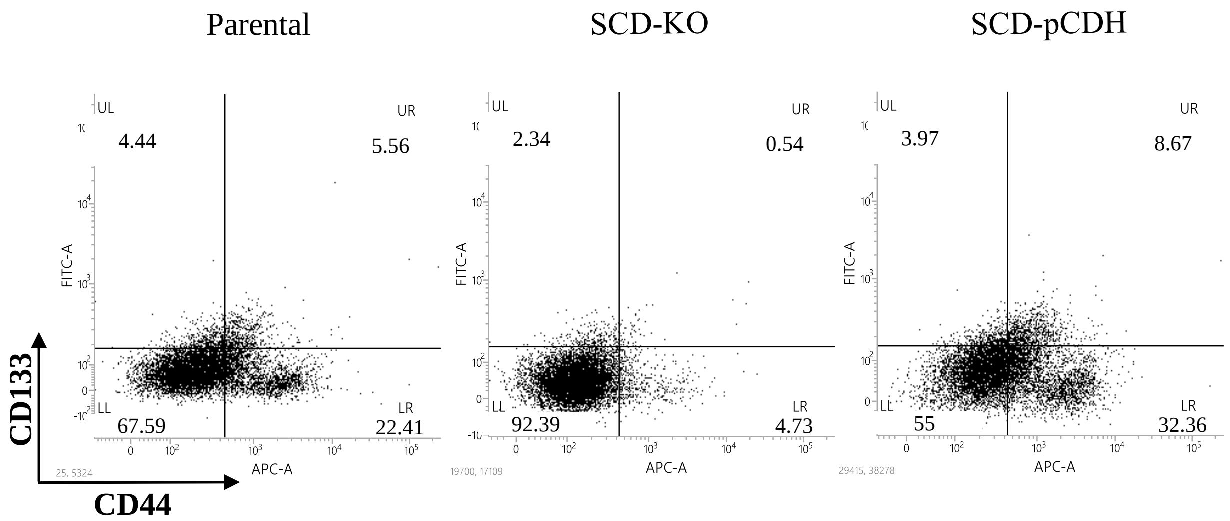

H.

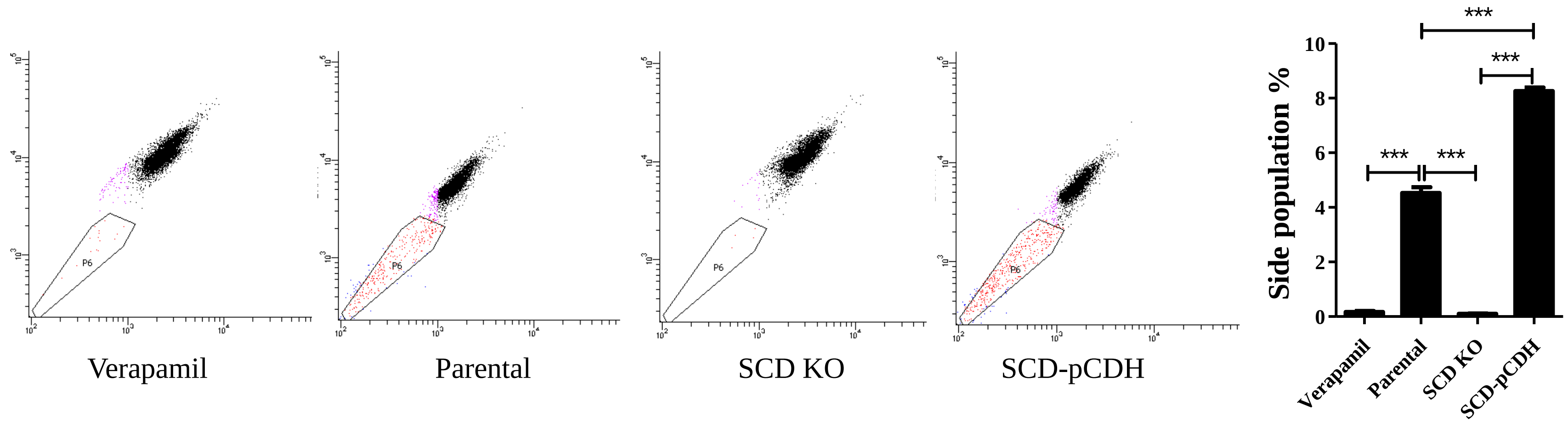

I.

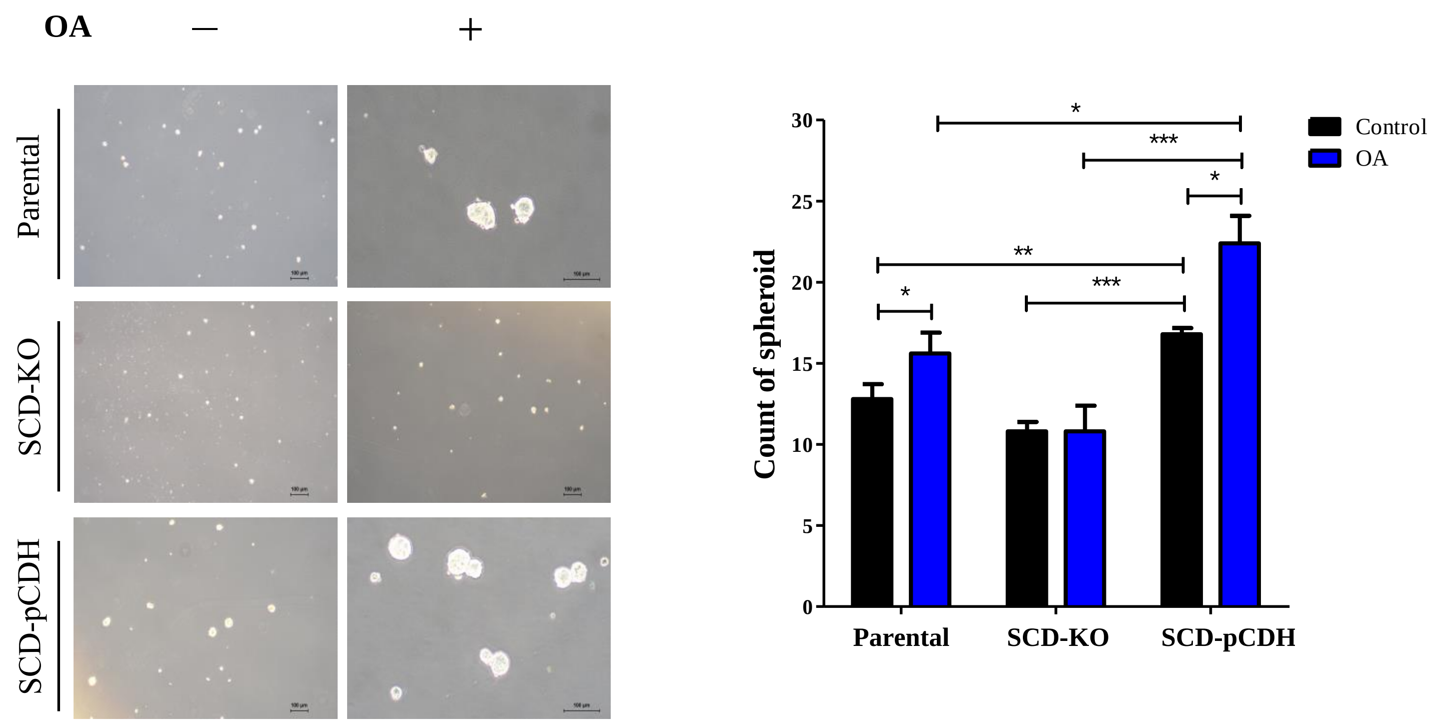

Additional Figure. S3

Additional

J.

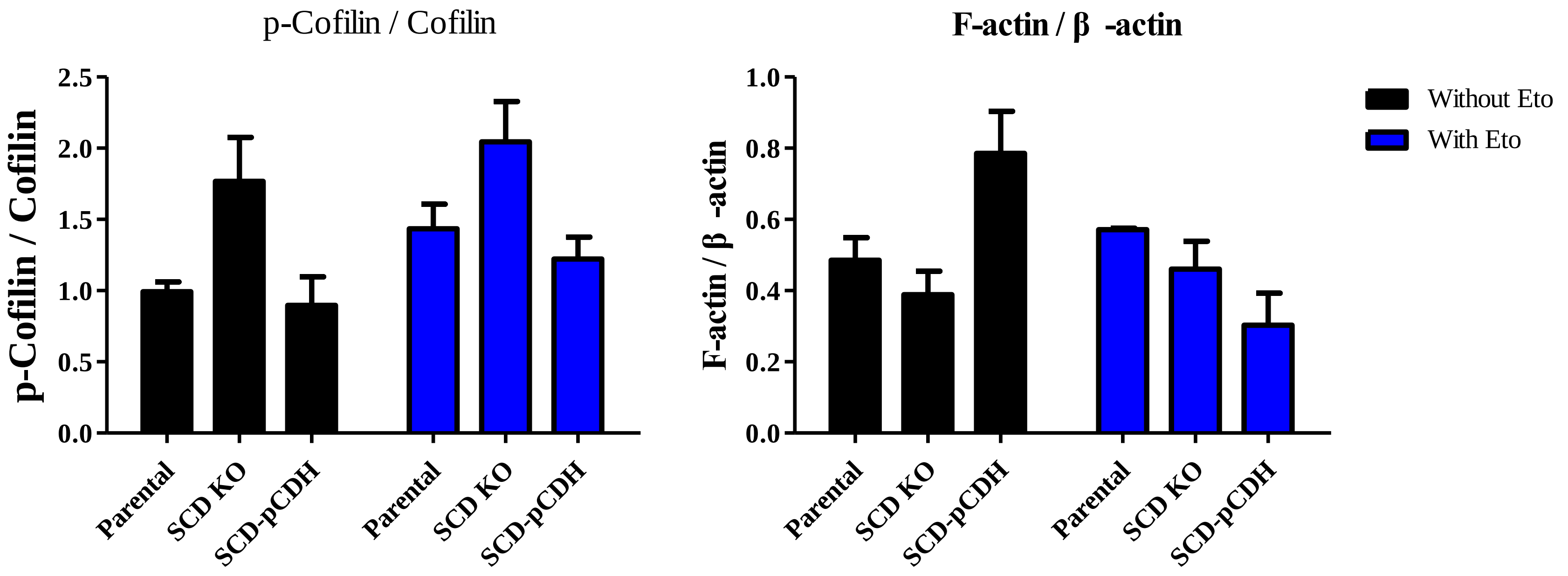

K.

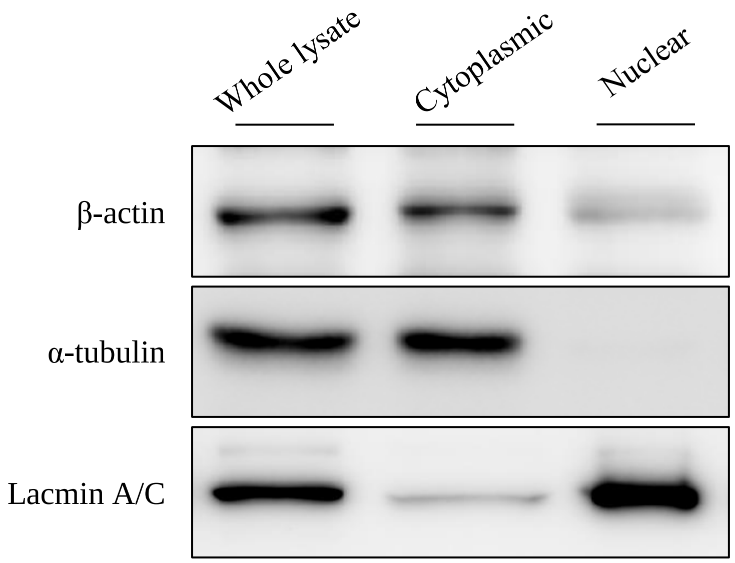

Additional Figure. S4

Additional

A.

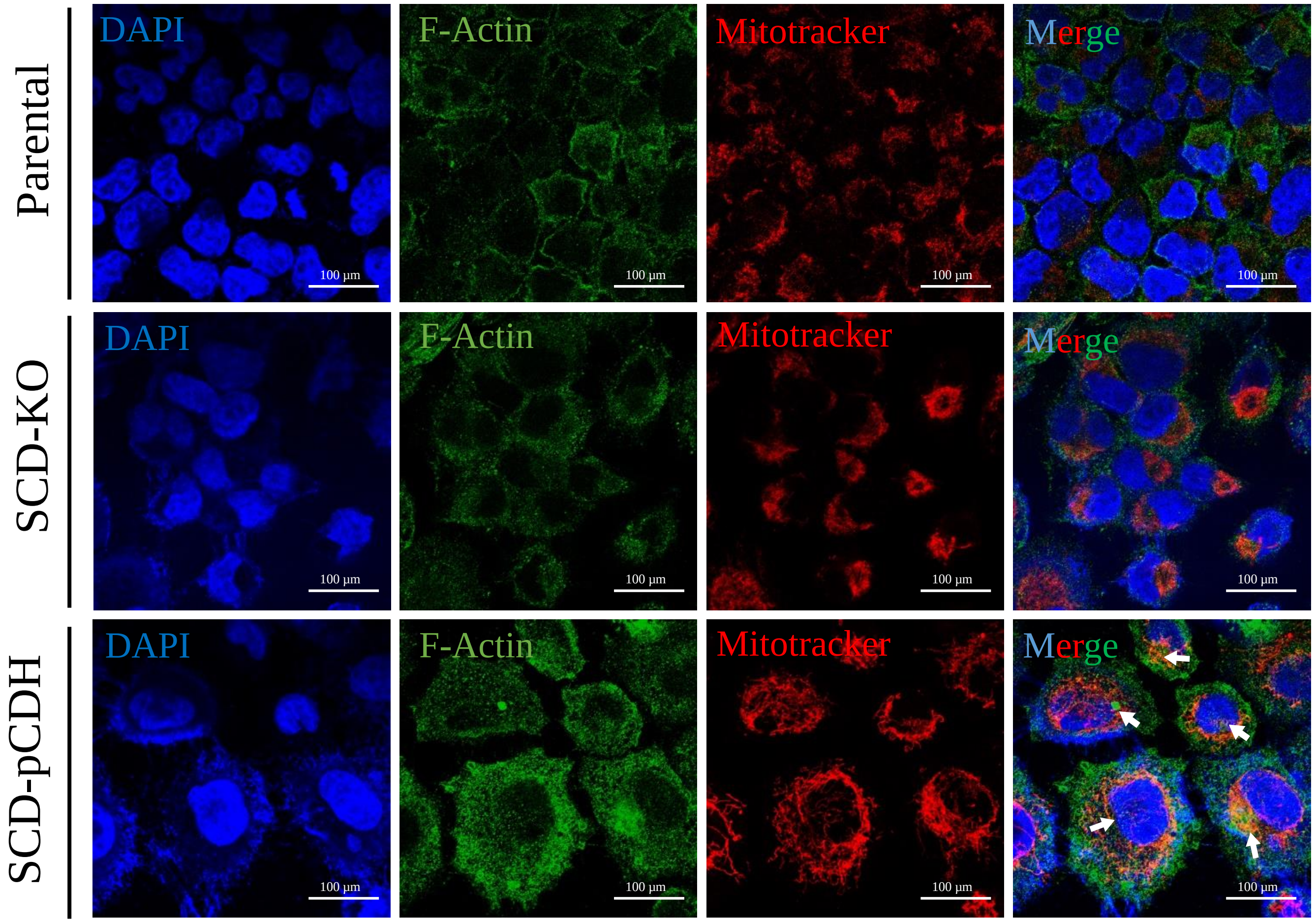

Additional Figure. S4

Additional

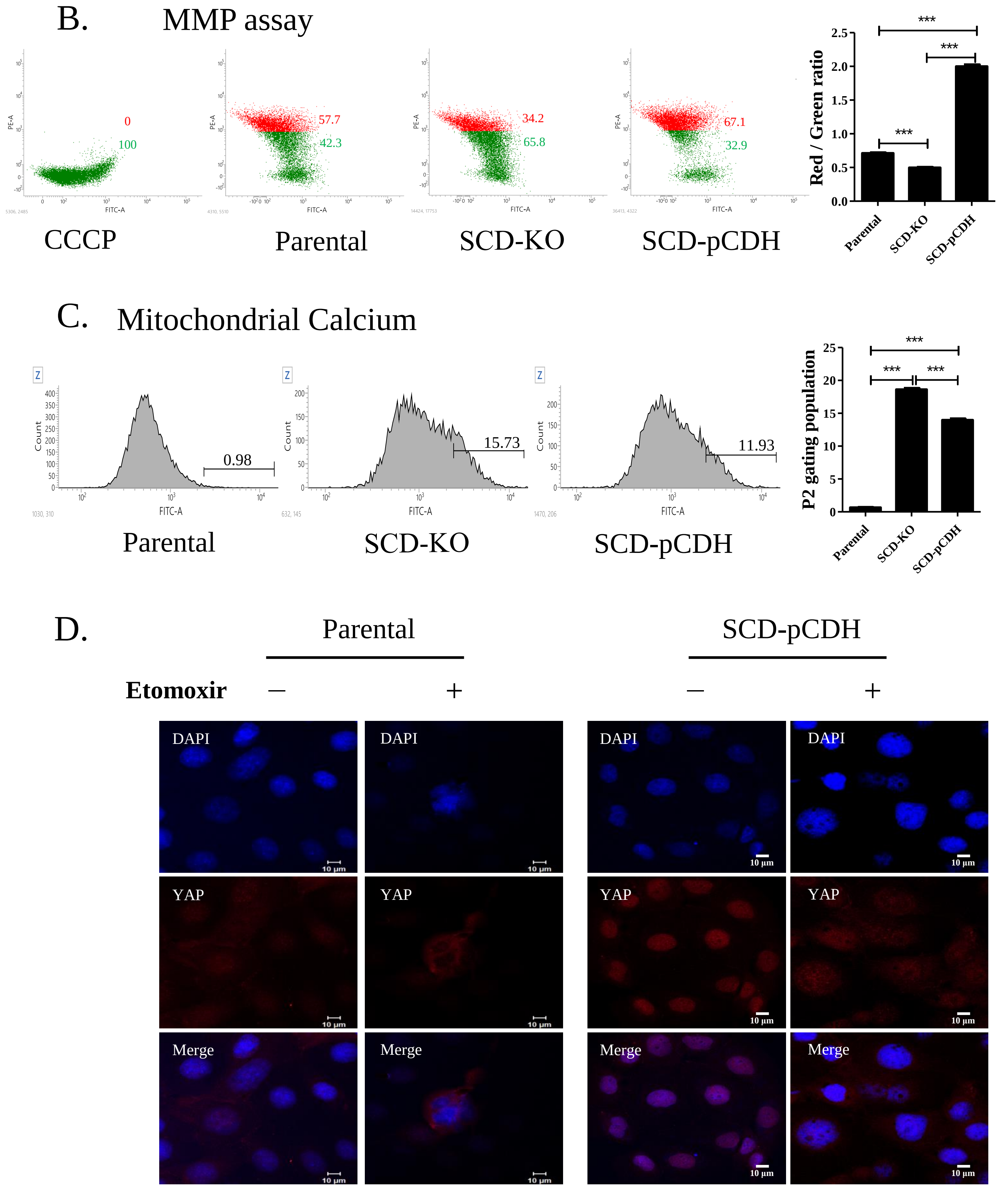

Additional Figure. S5

Additional

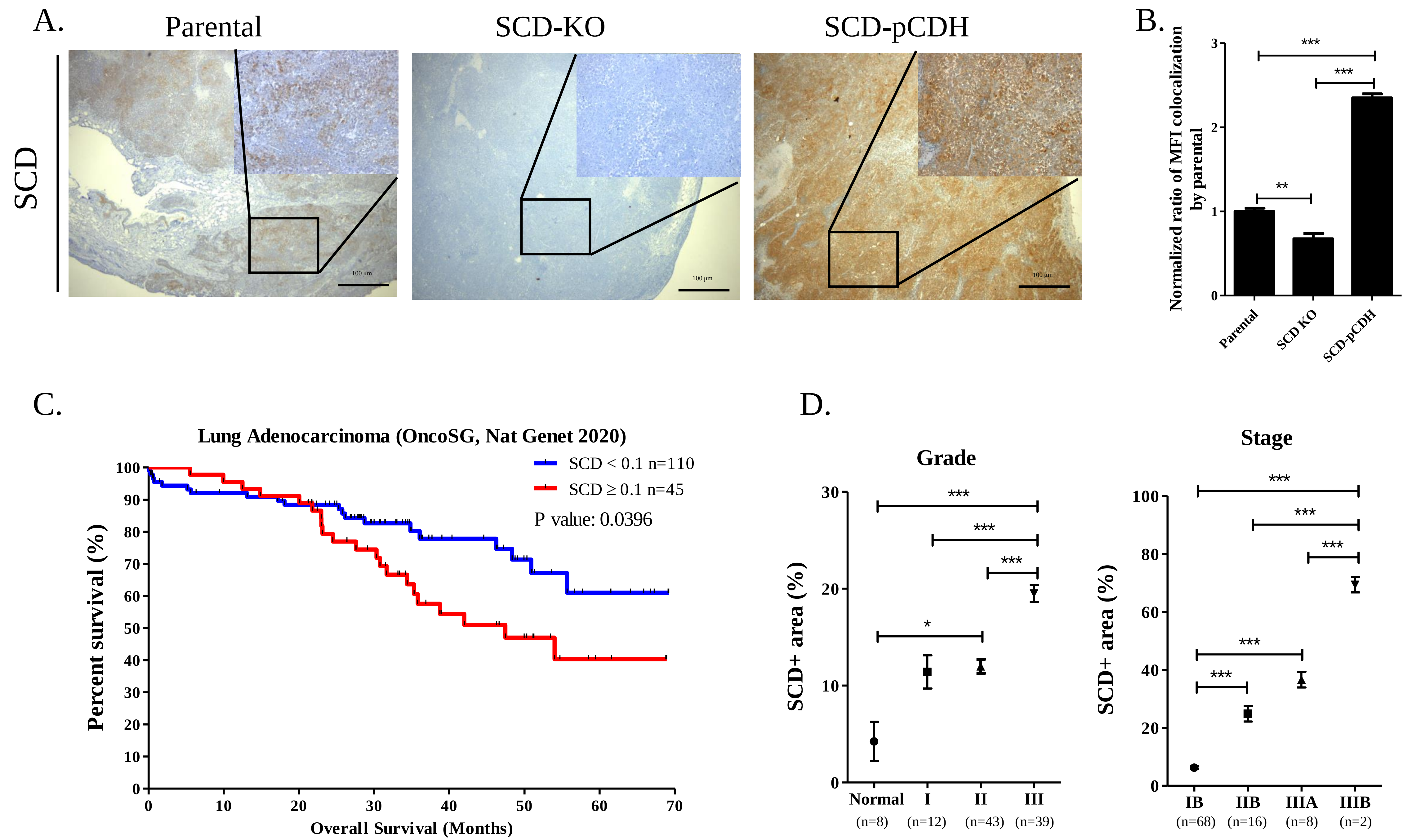

Supplement: Supplementary file 1 — Additional file 1. Figure S1. Phenotype of CAFs isolated from xenografted tumor masses (A) Staining for CD90 and FAP in H460 cells, NFs and CAFs. (B) Representative image of oil red O staining in a xenografted tumor. Red fluorescence indicates oil red O staining. 4× magnification. Scale bar: 150 μm. (C) Representative histogram for BODIPY staining in H460 cells, CAFs and NFs. The P2 population was established using H460 cells. ***P < 0.001 vs. H460 cells. Data indicate the mean ± SEM (n = 3). (D) Profile of fatty acid contents in supernatant from control and 1 μM CAY10566-treated CAFs. *P < 0.05, **P < 0.01, and ***P < 0.001. Data represent the mean ± SEM (n = 3). (E) Transcription levels of ACLY, ACAC, and FASN in H460 cells incubated in complete medium with supernatant of NF or CAF.1 for one day and subsequently treated with CAY10566. *P < 0.05. Data represent the mean ± SEM (n = 4). Figure S2. Effect of glucose deficiency on lipid metabolism and autophagosome. (A) Representative fluorescence image of BODIPY staining in H460 cells incubated with NF or CAF supernatant in complete or glucose-deficient medium for one day. 20× magnification. Scale bar: 50 μm. (B) Assessment of FFA levels in H460 cells incubated with supernatant of NF or CAF in complete or glucose-deficient medium for one day. $$P < 0.01, $$$P < 0.0001 vs. treatment of H460 cells with H460 supernatant. #P < 0.05, ###P < 0.0001 vs. treatment of glucose starved H460 cells with H460 supernatant. **P < 0.01 and ***P < 0.001. Error bars indicate SEM (n = 4). (C) Transcription levels of SREBP-1c and SCD in H460 cells incubated with supernatant of NF or CAF and cultured in complete or glucose-deficient medium for one day. #P < 0.05, ##P < 0.01, ###P < 0.001 vs. treatment of H460 cells with H460 supernatant. $$P < 0.01, $$$P < 0.001 vs. treatment of glucose starved H460 cells with H460 supernatant. *P < 0.05, **P < 0.01, and ***P < 0.001. Error bars indicate SEM (n = 3). (D) Transcription levels of SREBP-1c an [file 12935_2022_2824_MOESM1_ESM.pdf]
