# Supplementary material for: Oleic acid from cancer-associated fibroblast promotes cancer cell stemness by stearoyl-CoA desaturase under glucose-deficient condition
Source: Cancer Cell Int. 2022 Dec 13;22:404. doi: 10.1186/s12935-022-02824-3 (PMC9746202; doi:10.1186/s12935-022-02824-3)
Supplement: Supplementary file 2 — Additional file 2. Table S1. SCD primer sequences and single guide RNA. [file 12935_2022_2824_MOESM2_ESM.docx]

| ACAC | Forward | 5’-CTG GCT GCA TCC ATT ATG TCA-3’ |
| --- | --- | --- |
|  | Reverse | 5’-TGG TAG ACT GCC CGT GTG AA-3’ |
| ACLY | Forward | 5’-TCA CAT GAC GGC CAT TGT GG-3’ |
|  | Reverse | 5’-GCT GGC TTG GCT TTC TTT GC-3’ |
| FASN (Mouse) | Forward | 5’-TTG CTG GCA CTA CAG AAT GC-3’ |
|  | Reverse | 5’-AAC AGC CTC AGA GCG ACA AT-3’ |
| GAPDH (Mouse) | Forward | 5’-CAT CAC TGC CAC CCA GAA GAC TG-3’ |
|  | Reverse | 5’-ATG CCA GTG AGC TTC CCG TTC AG-3’ |
| FASN (Human) | Forward | 5’-AAG GAC CTG TCT AGG TTT GAT GC-3’ |
|  | Reverse | 5’-TGG CTT CAT AGG TGA CTT CCA-3’ |
| CD36 | Forward | 5’-TCT TTC CTG CAG CCC AAT G-3’ |
|  | Reverse | 5’-AGC CTC TGT TCC AAC TGA TAG TGA-3’ |
| SLC27A1 | Forward | 5’-TGA CAG TCG TCC TCC GCA AGA A-3’ |
|  | Reverse | 5’-CTT CAG CAG GTA GCG GCA GAT C-3’ |
| SCD | Forward | 5’-ATG ACC CCA CCT ACA AGG AT-3’ |
|  | Reverse | 5’-CTA GCG TAC TCC CCT TCT CT-3’ |
| SREBP | Forward | 5’-GCG GAG CCA TGG ATT GCA C-3’ |
|  | Reverse | 5’-CTC TTC CTT GAT ACC AGG CCC-3’ |
| Nanog | Forward | 5’-AGT CCC AAA GGC AAA CAA CCC ACT TC-3’ |
|  | Reverse | 5’-TGC TGG AGG CTG AGG TAT TTC TGT CTC-3’ |
| Oct4 | Forward | 5’-GAC AGG GGG AGG GGA GGA GCT AGG-3’ |
|  | Reverse | 5’-CTT CCC TCC AAC CAG TTG CCC CAA AC-3’ |
| GAPDH (Human) | Forward | 5’-GTC TCC TCT GAC TTC AAC AGC G-3’ |
|  | Reverse | 5’-ACC ACC CTG TTG CTG TAG CCA A-3’ |
| SCD | sgRNA | GCCTTCCTTATCCTTGTAGGTGG |

**Additional file Table S1. primer sequences and SCD single guide RNA.**
